# Antifungal potential of marine bacterial compounds in inhibiting *Candida albicans* Yck2 to overcome echinocandin resistance: a molecular dynamics study

**DOI:** 10.3389/fphar.2024.1459964

**Published:** 2024-10-17

**Authors:** Mohammed Merae Alshahrani

**Affiliations:** Department of Clinical Laboratory Sciences, Faculty of Applied Medical Sciences, Najran University, Najran, Saudi Arabia

**Keywords:** Yck2 inhibitors, *C. albicans*, marine-derived compound, antifungal agents, molecular dynamics

## Abstract

*Candida albicans* (*C. albicans*), a common fungal pathogen, poses a significant threat to immunocompromised individuals, particularly due to the emergence of resistance against echinocandins, a primary class of antifungal agents. Yck2 protein, a key regulator of cell wall integrity and signaling pathways in *C. albicans,* was targeted to overcome this resistance. A virtual screening was used to identify Yck2 inhibitors from marine bacterial compounds. Further re-docking, molecular dynamics simulations, and various analyses such as root mean square deviation (RMSD), root mean square fluctuation (RMSF), hydrogen bonding, free binding energy calculations, and RG-RMSD-based free energy landscape were conducted to evaluate the efficacy and stability of the identified compounds. Among the compounds screened, CMNPD27166 and CMNPD27283 emerged as the most promising candidates, demonstrating superior binding affinities, enhanced stability, and favorable interaction dynamics with Yck2, surpassing both the control and other compounds in efficacy. In contrast, CMNPD19660 and CMNPD24402, while effective, showed lesser potential. These findings highlight the utility of computational drug discovery techniques in identifying and optimizing potential therapeutic agents and suggest that marine-derived molecules could significantly impact the development of novel antifungal therapies. Further experimental validation of the leading candidates, CMNPD27166 and CMNPD27283, is recommended to confirm their potential as effective antifungal agents against echinocandin-resistant *C. albicans* infections.

## 1 Introduction

*Candida albicans* (*C. albicans*) is a ubiquitous fungal pathogen commonly found in the human microbiota, usually coexisting harmlessly (d’Enfert et al., 2020). However, under certain conditions, it can become opportunistic, leading to various infections such as superficial mucosal afflictions and severe systemic candidiasis (Mayer et al., 2013). These infections are particularly prevalent in immunocompromised individuals, such as those with HIV/AIDS (Samaranayake, 1992), undergoing chemotherapy, or receiving organ transplants, and pose significant treatment challenges. Hospitals worldwide report frequent outbreaks, often linked to biofilm formation on medical devices, leading to persistent infections that are difficult to eradicate (Ramage et al., 2005). Understanding the genomic structure and functional mechanisms of *C. albicans* is crucial for developing targeted therapies (Lee et al., 2023a; Caplan, 2018). Genomically, *C. albicans* has a dynamic repertoire that facilitates its adaptability and pathogenicity. The emergence of drug resistance to existing antifungal compounds worsens this problem, leading to an urgent need for novel therapeutic strategies (Vitiello et al., 2023; Delgado et al., 2024). Among the antifungal classes, echinocandins have been a popular drug due to their efficacy in targeting the cell wall of the fungus wall by inhibiting β-1,3-D-glucan synthase (Delgado et al., 2024; Ahmady et al., 2024; Caplan et al., 2020). However, the increasing prevalence of echinocandin-resistant strains of *C. albicans* underscores the urgency for innovative approaches to overcome this resistance. The current advances in understanding fungal biology have highlighted the potential of targeting non-essential stress response pathways to increase the efficacy of existing antifungal agents and circumvent resistance mechanisms (Caplan et al., 2020; Iyer et al., 2022). Among the potential therapeutic targets, the non-essential Stress Kinase Yck2 is noteworthy (Lee et al., 2023a; Caplan et al., 2020; Rabaan et al., 2024). Yck2 plays a very important role in the yeast’s ability to adapt to environmental stresses, morphological changes, and survival under antifungal pressure (Caplan et al., 2020; Iyer et al., 2022; Jung et al., 2017; Liboro et al., 2021). The synergy between Yck2 inhibition and echinocandins has been well-documented in overcoming echinocandin resistance in *C. albicans*. Yck2, a key regulator of cell wall integrity, exacerbates cell wall stress responses when inhibited, enhancing echinocandins’ efficacy. This combination approach effectively restores drug sensitivity in resistant strains, supporting targeting stress kinases like Yck2 as a promising therapeutic strategy against antifungal resistance (Lee et al., 2023a; Caplan, 2018; Caplan et al., 2020; Iyer et al., 2022; Lee et al., 2023b). Current therapeutic strategies against Yck2 involve a limited number of inhibitors, which have shown efficacy in disrupting the pathogenic mechanisms of *C. albicans*. However, these inhibitors face significant challenges, including poor selectivity, limited bioavailability, and the rapid emergence of resistance. These limitations underscore the urgent need for enhanced efficacy and safety profiles for new inhibitors. The exploration of marine environments has unveiled a diverse array of microorganisms, including bacteria with potent bioactive compounds (Karthikeyan et al., 2022).

The significance of marine-derived bacterial compounds, previously identified as potential antifungal agents, has been increasingly recognized. These compounds, sourced from the diverse marine environment, offer promising solutions against drug-resistant fungal infections, showcasing their potential in developing new antifungal therapies. Recent studies have highlighted their unique mechanisms of action, which differ from traditional antifungal agents, making them effective against a broader range of fungal pathogens. Additionally, the vast biodiversity of marine ecosystems provides a rich source of novel compounds that could lead to the discovery of new classes of antifungal drugs. This underscores the importance of continued research and exploration of marine microorganisms in the fight against fungal diseases (Lu et al., 2014). These metabolites have demonstrated various biological activities, offering a promising pool of novel molecular frameworks for drug discovery. This study was designed to harness the potential of the marine bacterial compound database to discover new Yck2 inhibitors using an advanced computational drug discovery approach. Our methodology integrated several computational techniques: virtual screening for the selection of drug-like molecules, re-docking to confirm the stability and appropriateness of ligand binding, and molecular dynamics (MD) simulations to understand the dynamical behavior of the enzyme-inhibitor complexes. The free-binding energy calculations were performed, and an RG-RMSD-based free energy landscape was used to evaluate the potential efficacy of the candidates under physiologically relevant conditions.

Through this comprehensive computational approach, this study focused on identifying potent inhibitors of Yck2 that can enhance the efficacy of echinocandins, restoring their antifungal activity against resistant strains and contributing to developing new antifungal treatments by targeting the stress kinase. The successful identification and characterization of such inhibitors could significantly impact the management of fungal infections, particularly in vulnerable patient populations. Integrating marine-derived bacterial compounds with advanced computational screening techniques presents a novel pathway for discovering antifungal agents. Our study expands the understanding of marine bacteria as a resource for therapeutic agents and showcases the potential of computational methods in identifying promising candidates against challenging biological targets like Yck2. This approach sets the foundation for further experimental validation and development of effective antifungal therapies, aiming to address the critical issue of antifungal resistance.

## 2 Methodology

### 2.1 Data collection, virtual screening and pharmacokinetics study

The initial phase of our study involved a detailed computational screening process to identify potential inhibitors of Yck2, a key regulatory protein in *C. albicans*. The protein and ligand datasets were prepared meticulously to ensure the accuracy of our computational predictions. The crystal structure of Yck2 was retrieved from the Protein Data Bank (PDB) (Berman et al., 2000), with the PDB ID 6U6A selected for its resolution and relevance (Caplan et al., 2020). Before docking, the protein was prepared using the Chimera Dock Prep utility (Pettersen et al., 2004). It involved cleaning the protein structure by removing water molecules, adding hydrogen atoms, and correcting missing side chains to stabilize the protein’s active conformation. The marine bacterial compounds from the Comprehensive Marine Natural Products Database were downloaded (Lyu et al., 2021). MtiOpenScreen web server was utilized for virtual screening (Labbé et al., 2015) Integrating Lipinski filter checks will refine the selection process. This step was crucial for eliminating compounds with poor pharmacokinetic properties from the screening funnel. Based on their docking scores, four were selected from a vast library of compounds, indicating a high affinity and specificity for the binding site of Yck2. These compounds were then subjected to a rigorous re-docking process to validate the initial results (Ferreira et al., 2015). Re-docking was executed using the AutoDock Vina plugin in Chimera, ensuring a refined analysis of ligand interactions (Trott and Olson, 2010). Each compound was docked into the active site of the prepared Yck2 protein model, with the process repeated several times to ensure consistency. A control molecule (Q0J), known to inhibit Yck2, was docked under identical conditions as a benchmark for assessing the relative binding efficiencies of the new compounds. Also, the drug metabolism and pharmacokinetic properties of these selected compounds were studied using the SwissADME and ProTox 3.0 web servers (Daina et al., 2017; Banerjee et al., 2018).

### 2.2 Dynamical analysis

The molecular MD simulations and subsequent trajectory analyses were conducted to explore further the binding stability and dynamic behavior of potential inhibitors identified from virtual screening. This phase focused on five complexes for the simulations under the influence of Generalized Amber Force Field (GAFF) (Wang et al., 2004) within the AMBER software suite (Case et al., 2005).

#### 2.2.1 Ligand preparation and system building

Initially, each ligand was prepared using the Antechamber tool within AMBER (Wang et al., 2006), which facilitated the generation of molecular topologies and the assignment of partial atomic charges necessary for the force field. This preparation ensured that all ligands were optimized for the simulation environment. Following ligand preparation, the LEaP tool of AMBER was utilized to construct the full simulation system (Case et al., 2005). This involved integrating the prepared Yck2 protein, optimized ligands, and solvent model (TIP3P) (Mark and Nilsson, 2001). The neutralized step was performed by adding counter ions to balance the charge, ensuring a realistic simulation environment.

#### 2.2.2 MD simulation setup, minimization, and production run

Each system was subjected to energy minimization through Pmemd module to eliminate any steric clashes or unusual geometry arising from the system setup (Salomon-Ferrer et al., 2013; Alobaida et al., 2024). The MD simulations were performed using the AMBER simulation package, where each system was first minimized by the Pmem. cuda module (Case et al., 2005) and then gradually heated to 300 K, followed by equilibration under constant pressure and temperature conditions. The Langevin thermostat was employed for temperature regulation (Farago, 2019), while pressure was managed by Berendsen barostat (Lin et al., 2017). The production phase of the MD simulation was carried out over 300 ns, providing adequate time to observe the behavior of the ligand-protein interactions under dynamic conditions.

#### 2.2.3 Trajectory analysis

The trajectories generated during the MD simulations were analyzed using the cpptraj tool of AMBER suite (Roe and Cheatham, 2013). Key metrics assessed included root mean square deviation (RMSD) to evaluate the stability of the protein-ligand complexes, and root mean square fluctuation (RMSF) to examine the flexibility of specific protein residues upon ligand binding. Additionally, the Radius of Gyration (RG) and potential energy were monitored to assess the compactness and energetic stability of the complexes throughout the simulation. While the RG-RMSD based free energy landscape was also constructed for each complex by the geo-measure plugin in pymol software (Kagami et al., 2020). This analysis helped in identifying the most stable conformations and the energy barriers between different states, providing information about the dynamic behavior of the inhibitors within the binding site of Yck2. The MD simulation with rigorous trajectory analysis provided a comprehensive evaluation of the binding efficacy and stability of the identified inhibitors.

### 2.3 Free binding energy analysis

To ascertain the binding affinities of potential inhibitors against the Yck2 protein, the free binding energy calculations were conducted using the MMPBSA.py tool (Miller et al., 2012), a component of the Amber software suite (Case et al., 2005). This process began by extracting the last 50 nanoseconds of the molecular dynamics simulation trajectories, which provides a representative sample of the system’s stable interactions. The MMPBSA.py methodology involves the computation of MMGBSA. This approach quantifies the free energy of binding between the inhibitor and the protein by considering electrostatic and van der Waals interactions, solvation effects, and the entropy contribution of binding. Specifically, the total binding energy is derived from the difference in free energy between the bound state and the sum of the free energies of the unbound receptor and ligand. By integrating these calculations, It can estimate the binding affinity, which is crucial for identifying the most promising compounds. This detailed energy analysis helps pinpoint interactions vital for strong and specific binding, thereby guiding the selection of potential candidates for further experimental validation.

### 2.4 RG-RMSD-based free energy calculation

To elucidate the conformational stability and binding efficiency of the Yck2 inhibitors derived from marine bacterial compounds, an RG-RMSD-based Free Energy Landscape analysis was employed (Duan et al., 2019). This analytical approach was executed using the Geo-measure plugin within the PyMOL molecular visualization system (Kagami et al., 2020). The methodology commenced with the generation of MD simulations for each of the four selected complexes, conducted using the Amber simulation suite (Case et al., 2005). These simulations provided detailed atomic motion data over time, capturing the dynamic interactions between the compounds and the Yck2 protein. Following the simulations, RG-RMSD calculations were performed. This process involves measuring the RMSD of the atomic positions from a reference structure (typically the starting model) to assess structural deviations over the course of the simulation. Concurrently, the RG was computed for each frame to determine the protein-ligand complex’s compactness, which indicates its structural integrity under dynamic conditions. The combined RG-RMSD data points were then plotted to create a free-energy landscape (Homeyer and Gohlke, 2012). This landscape provides a visual representation of the energy barriers and wells encountered by the complex during the simulation, reflecting the most stable conformations and the transitions between them. Such a landscape aids in identifying the most energetically favorable interactions between the compound and the Yck2 protein, offering insights into the potential efficacy of the inhibitors. This methodological approach thus serves as a crucial tool in the computational evaluation of drug candidates.

## 3 Results

### 3.1 Virtual screening and ADME&T analysis

Virtual screening is an effective computational strategy to search for bioactive molecules within large chemical libraries, targeting those that demonstrate potential binding affinity to specific biological receptors (Li et al., 2020). The virtual screening functionality on the MTiopen screen web server was used to identify promising candidates from the Comprehensive Marine Natural Products Database (Lyu et al., 2021; Labbé et al., 2015). This selection was based on the binding energy values recorded during the screening process and conducted a comprehensive examination of 2,895 compounds from this database, where the binding energies ranged from −12.7 kcal/mol to −7.3 kcal/mol for the top 1,500 compounds, as detailed in Supplementary Table S1. Four compounds stood out due to their significant binding energies: CMNPD27283, CMNPD19660, CMNPD27166, and CMNPD24402.

The absorption, distribution, metabolism, excretion (ADME), and toxicity (T) analysis of these four compounds were also analyzed (Table 1). Based on the results obtained, CMNPD19660 was water-soluble, and CMNPD27283 exhibited moderate solubility. However, CMNPD27166 and CMNPD24402 are poorly soluble. The human gastrointestinal absorption of the CMNPD27283, CMNPD19660, and CMNPD24402 are high, suggesting that these compounds can be effectively taken up into the bloodstream when administered orally. However, the CMNPD27166 compound showed low human gastrointestinal absorption. All the compounds exhibited negative results for blood-brain barrier (BBB) permeation. The CMNPD27283 and CMNPD27166 exhibited moderate toxicity, whereas CMNPD19660 and CMNPD24402 were nontoxic compounds.

**TABLE 1:** ADME and T analysis of the selected marine bacterial compounds.

Molecule	Naseseazine C (CMNPD27283)	Dermacozine E (CMNPD19660)	Wailupemycin H (CMNPD27166)	Metagenetriindole a (CMNPD24402)
MW	564.63	396.4	722.69	389.45
#Rotatable bonds	3	2	6	4
#H-bond acceptors	4	4	11	1
#H-bond donors	3	2	6	3
MR	172.21	116.43	199.33	121.18
TPSA	117.85	110.84	198.87	64.44
iLOGP	3.17	2.1	3.35	2.47
XLOGP3	2.68	1.48	6.73	5.61
WLOGP	−0.25	2.69	6.7	6.15
MLOGP	1.11	2.16	2.33	3.1
Silicos-IT Log P	1.99	3.8	7.29	6.65
Consensus Log P	1.74	2.45	5.28	4.79
ESOL Log S	−5.1	−3.69	−8.71	−6.19
ESOL Solubility (mg/mL)	4.53E-03	8.09E-02	1.40E-06	2.51E-04
ESOL Solubility (mol/L)	8.03E-06	2.04E-04	1.94E-09	6.44E-07
ESOL Class	Moderately soluble	Soluble	Poorly soluble	Poorly soluble
Ali Log S	−4.81	−3.41	−10.71	−6.73
Ali Solubility (mg/mL)	8.80E-03	1.53E-01	1.41E-08	7.32E-05
Ali Solubility (mol/L)	1.56E-05	3.85E-04	1.94E-11	1.88E-07
Ali Class	Moderately soluble	Soluble	Insoluble	Poorly soluble
Silicos-IT LogSw	−7.15	−7.71	−12.97	−10.02
Silicos-IT Solubility (mg/mL)	3.96E-05	7.64E-06	7.78E-11	3.74E-08
Silicos-IT Solubility (mol/L)	7.01E-08	1.93E-08	1.08E-13	9.62E-11
Silicos-IT class	Poorly soluble	Poorly soluble	Insoluble	Insoluble
GI absorption	High	High	Low	High
BBB permeant	No	No	No	No
Pp substrate	Yes	Yes	Yes	Yes
CYP1A2 inhibitor	No	No	No	Yes
CYP2C19 inhibitor	No	No	No	No
CYP2C9 inhibitor	No	Yes	No	No
CYP2D6 inhibitor	Yes	No	No	No
CYP3A4 inhibitor	Yes	No	No	No
log Kp (cm/s)	−7.84	−7.67	−5.93	−4.69
Lipinski #violations	1	0	3	0
Ghose #violations	3	0	4	1
Veber #violations	0	0	1	0
Egan #violations	0	0	2	1
Muegge #violations	1	0	6	1
Bioavailability Score	0.55	0.55	0.17	0.55
PAINS #alerts	0	0	0	0
Brenk #alerts	0	2	0	0
Leadlikeness #violations	1	1	2	2
Synthetic Accessibility	5.23	2.91	6.14	2.92
Toxicity	Moderate toxicity	Non-toxic	Moderate toxicity	Non- toxic

### 3.2 Redocking and interaction analysis

Re-docking is an essential process in drug development, involving a meticulous reassessment of the interactions between ligand molecules and receptor proteins (Bhardwaj and Thounaojam, 2024). In our research, the specific parameters were applied to the re-docking process: the docking grid’s center was established at coordinates (X = 18.67, Y = −14.47, Z = 14.74) with each axis (X, Y, Z) spanning 20 Å. This method is critical for confirming initial docking results and elucidating crucial interactions with the ligand at the receptor’s binding site. Each ligand was precisely aligned with the target protein during the re-docking and assessed relative to a benchmark molecule, resulting in at least nine distinct poses for each ligand-receptor pair. The pose exhibiting the most favorable docking energy, identified by the lowest negative value, was selected for detailed analysis. This phase is pivotal for further examining the stability of the complex and the affinity between the ligand and the target protein. Concurrently, a virtual screening was performed to investigate a broad library of medicinal compounds. This identified four compounds with notably significant binding energies, recorded as −12.8 kcal/mol, −12.1 kcal/mol, −11.9 kcal/mol, and −12.0 kcal/mol, designated as compounds CMNPD27283, CMNPD19660, CMNPD27166, and CMNPD24402. These compounds were chosen for further investigation. A control molecule, “Q0J,” with a binding energy of −10.5 kcal/mol, served as a comparison standard.

**FIGURE 1:**
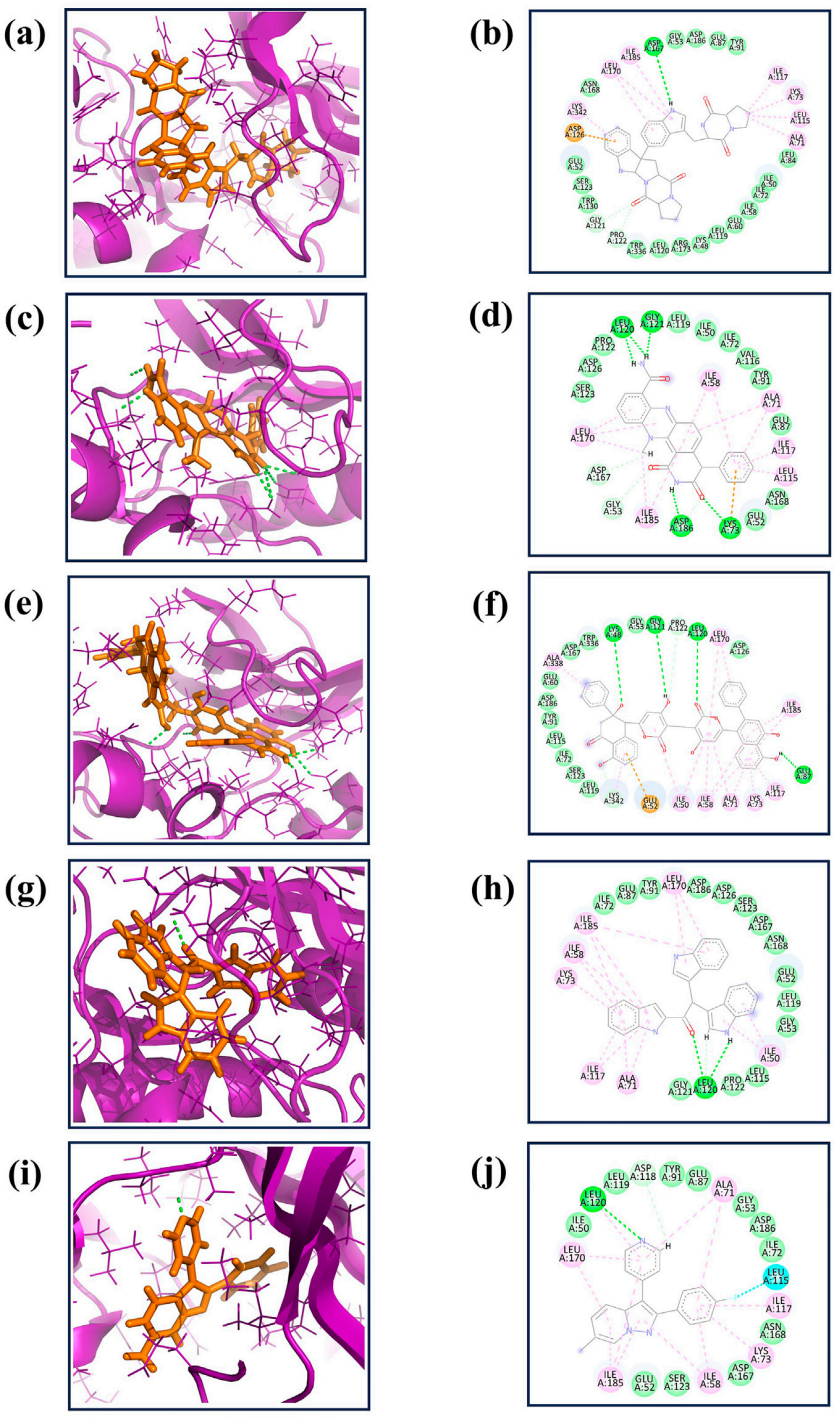
The 3D interaction diagram of the selected marine compounds complexed with Yck2 protein. **(A, B)** CMNPD27283, **(C, D)** CMNPD19660, **(E, F)** CMNPD27166, and **(G, H)** CMNPD24402 and **(I, J)** control (Q0J). Herein, in the 3D representation, the ligand structures are represented in orange colour and the protein backbone in violet colour. In the 2D interactions, the green colour bonds represent the hydrogen bonds, the green colour leaflets denote hydrophobic bond-forming residues, and the pink colour leaflets and bonds denote the Pi-pi stacking/pi-alkyl interaction.

As detailed in Table 2, our study comprehensively analyzed the molecular interactions between selected compounds and a target protein. The three-dimensional structures of these molecules, including the control, were visualized using PyMOL software (DeLano, 2002), while two-dimensional figures were created using Biodiscovery Studio (BIOVIA, Dassault Systèmes, 2020). These visualizations facilitated a deeper understanding of their spatial conformations and interactions within the binding site. Compound CMNPD27283 exhibited a singular hydrogen bond with the residue Asp167 and engaged in sixteen hydrophobic interactions with residues including Gly53, Asp186, Glu87, Tyr91, Leu84, Ile50, Ile72, Ile58, Glu60, Leu119, Lys48, Arg173, Leu120, Trp130, Ser123, and Glu52. Additionally, this compound formed seven pi-alkyl bonds with residues such as Lys185, Leu170, Lys342, Ile117, Lys73, Leu115, and Ala71 (Figures 1A, B). In contrast, compound CMNPD19660 formed four hydrogen bonds with residues like Leu120, Gly121, Lys73, and Asp167, and exhibited eleven hydrophobic interactions with residues including Leu119, Ile50, Ile72, Val116, Tyr91, Glu87, Asn168, Glu52, Ser123, Asp126, and Pro122. It also formed six pi-alkyl interactions with residues such as Ala71, Ile117, Leu115, Ile58, Ile185, and Leu (Figures 1C, D). Compound CMNPD27166 established four hydrogen bonds with residues Lys48, Gly121, Leu120, and Glu87. It also formed twelve hydrophobic interactions with residues such as Leu119, Ser123, Ile72, Leu115, Tyr91, Asp186, Glu60, Asp167, Trp336, Gly53, Pro122 and Asp126, and had eight pi-alkyl interactions with residues including Leu170, Ile185, Ile117, Lys73, Ala71, Ile58, Ile50, and Ala338 (Figures 1E, F). Compound CMNPD24402, on the other hand, formed a single hydrogen bond with Leu120, fourteen hydrophobic interactions with residues such as Ile72, Glu87, Tyr91, Asp186, Asp126, Ser123, Asp167, Asn168, Glu52, Leu119, Gly53, Leu115, Pro122 and Gly121, and participated in seven pi-alkyl interactions with residues like Lys73 and Ile58 (Figures 1G, H). The control compound displayed a similar pattern of interactions, forming one hydrogen bond with Leu120, ten hydrophobic interactions with residues including Ile50, Leu119, Tyr91, Glu87, Gly53, Asp186, Ile72, Asp167, Ser123, Glu52, and six pi-alkyl bonds with residues such as Leu170, Ala71, Ile117, Lys73, ile58, and Ile185 (Figures 1I, J). This comprehensive profiling highlights the specific and varied binding interactions contributing to these compounds’ molecular docking and stability with the target protein.

**TABLE 2:** The intermolecular analysis of four selected ligand-protein complexes (a) CMNPD27283, (b) CMNPD19660, (c) CMNPD27166, and (d) CMNPD24402 and (e) control (Q0J).

S.No	Compounds	Hydrogen bonds	Hydrophobic interaction	Pi-pi stacking/pi-alkyl interaction
1	CMNPD27283	Asp167	Gly53, Asp186, Glu87, Tyr91, Leu84, Ile50, Ile72, Ile58, Glu60, Leu119, Lys48, Arg173, Leu120, Trp130, Ser123, and Glu52	Lys185, Leu170, Lys342, Ile117, Lys73, Leu115, and Ala71
2	CMNPD19660	Leu120, Gly121, Lys73 and Asp167	Leu119, Ile50, Ile72, Val116, Tyr91, Glu87, Asn168, Glu52, Ser123, Asp126, and Pro122	Ala71, Ile117, Leu115, Ile58, Ile185 and Leu
3	CMNPD27166	Lys48, Gly121, Leu120 and Glu87	Leu119, Ser123, Ile72, Leu115, Tyr91, Asp186, Glu60, Asp167, Trp336, Gly53, Pro122 and Asp126	Leu170, Ile185, Ile117, Lys73, Ala71, Ile58, Ile50, and Ala338
4	CMNPD24402	Leu120	Ile72, Glu87, Tyr91, Asp186, Asp126, Ser123, Asp167, Asn168, Glu52, Leu119, Gly53, Leu115, Pro122 and Gly121	Lys73, Ile58, Ile185, Leu170, Ile50, Ala71 and Ile117
5	Control	Leu120	Ile50, Leu119, Tyr91, Glu87, Gly53, Asp186, Ile72, Asp167, Ser123, Glu52	Leu170, Ala71, Ile117, Lys73, Ile 58, and Ile185

### 3.3 MD simulation

**FIGURE 2:**
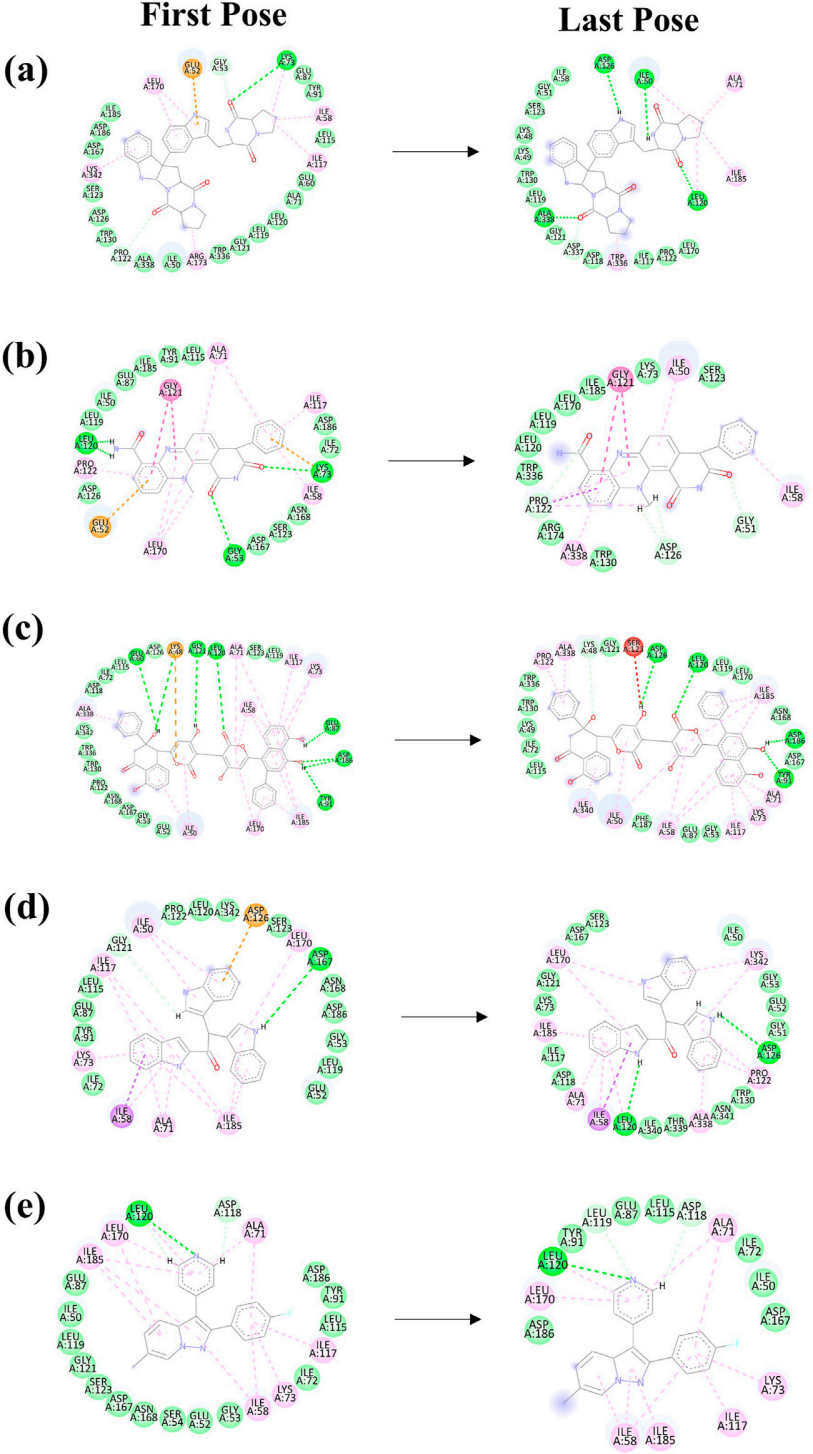
The 2D interaction diagram of the first and last pose of the Yck2-marine complexes viz., **(A)** CMNPD27283, **(B)** CMNPD19660, **(C)** CMNPD27166, and **(D)** CMNPD24402 and **(E)** control (Q0J) extracted from the 300 ns simulation trajectory. In the 2D interactions, the green colour bonds represent the hydrogen bonds, the green colour leaflets denote hydrophobic bond-forming residues, and the pink colour leaflets and bonds denote the Pi-pi stacking/pi-alkyl interaction.

MD simulations are pivotal for understanding the dynamic stability of protein-ligand complexes, providing in-depth views of molecular interactions over time (Bowers et al., 2006). This study conducted a 300-nanosecond MD simulation to investigate the binding interactions and mechanistic dynamics of four selected compounds with a target protein. The extended duration of the simulation ensures that the system reaches equilibrium and that the observed interactions are representative of the true behavior of the protein-ligand complexes. Throughout the simulation, the trajectories of each protein-ligand complex were closely monitored. This involves tracking the positions and movements of all atoms in the system, providing a comprehensive picture of how the complexes behave over time. The strength of the interaction between the protein and the ligand was assessed. Changes in the shape and structure of the protein were observed. These shifts can reveal how the protein adapts to ligand binding and can affect the protein’s functionality. The energy landscape of the protein-ligand complex was analyzed to understand the stability of the interactions. Figures 2A–E illustrates the first and last poses of the MD simulation for each protein-ligand complex. These snapshots visually represent the conformational changes and binding interactions that occurred during the simulation. The first pose represents the protein-ligand complex’s initial configuration at the simulation’s start. It serves as a reference point for observing changes over time. The last pose shows the final configuration of the complex after 300 nanoseconds. Comparing this with the first pose highlights the conformational shifts and stabilization during the simulation.

#### 3.3.1 RMSD analysis

**FIGURE 3:**
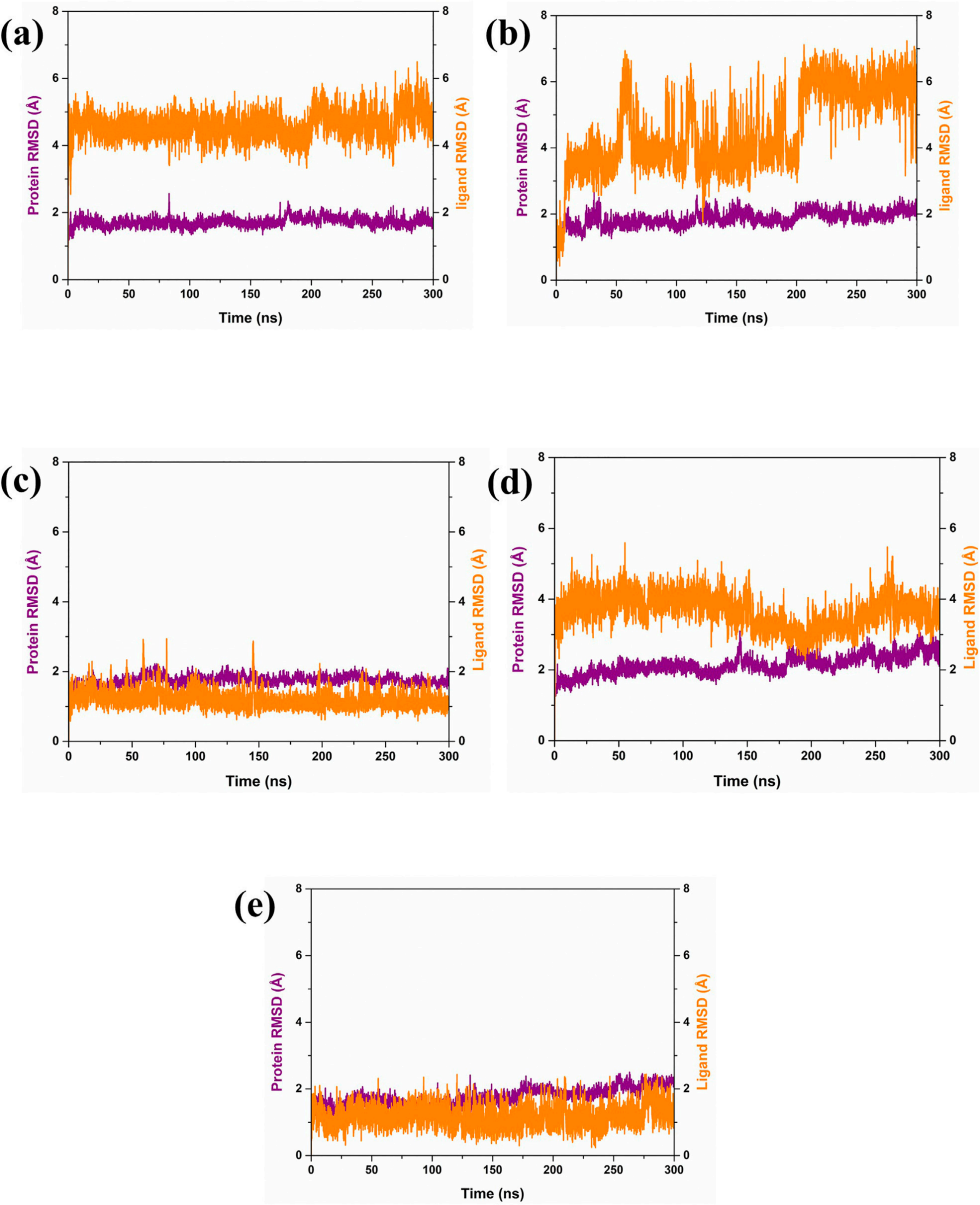
RMSD plot of Yck2-marine complexes viz., **(A)** CMNPD27283, **(B)** CMNPD19660, **(C)** CMNPD27166, and **(D)** CMNPD24402 and **(E)** control (Q0J). Herein, the protein RMSD is represented in violet colour, and ligand RMSD is represented in orange colour.

In our analysis of protein-ligand complexes, compounds CMNPD27283 and CMNPD27166 demonstrated protein root mean square deviation (RMSD) values of less than 2 Å, indicating a high level of structural stability within the bound state (Figures 3A, C). The protein RMSD of the CMNPD19660 and control compound showed RMSD values greater than or equal to 2 Å, suggesting the structural stability of the protein during the simulation (Figures 3B, E). Altogether, the protein displayed structural stability, due to binding the selected marine compounds throughout the simulation. For ligand, CMNPD27283 maintained a ligand RMSD value of around 5 Å (Figure 3A). The compound CMNPD19660 showed fluctuations, reached up to 7 Å, and remained in the equilibrium stage from 220 ns to 300 ns (Figure 3B). The CMNPD27166 compound also fluctuated but in an acceptable range of 4 Å (Figure 3C). The CMNPD24402 presented a ligand RMSD of less than 4 Å, indicating relatively stable binding (Figure 3D). The control compound exhibited a consistent and stable RMSD of less than 2 Å throughout the simulation period (Figure 3E). The ligand RMSD analysis showed that the CMNPD24402 showed maximum stability similar to the control compound, and the CMNPD19660 displayed minimum stability. However, the compound remained in the binding site, indicating that the ligand may have changed the binding pattern during the simulation. Also, the other two ligands, CMNPD27283 and CMNPD27166, showed acceptable binding stability.

#### 3.3.2 RMSF analysis

**FIGURE 4:**
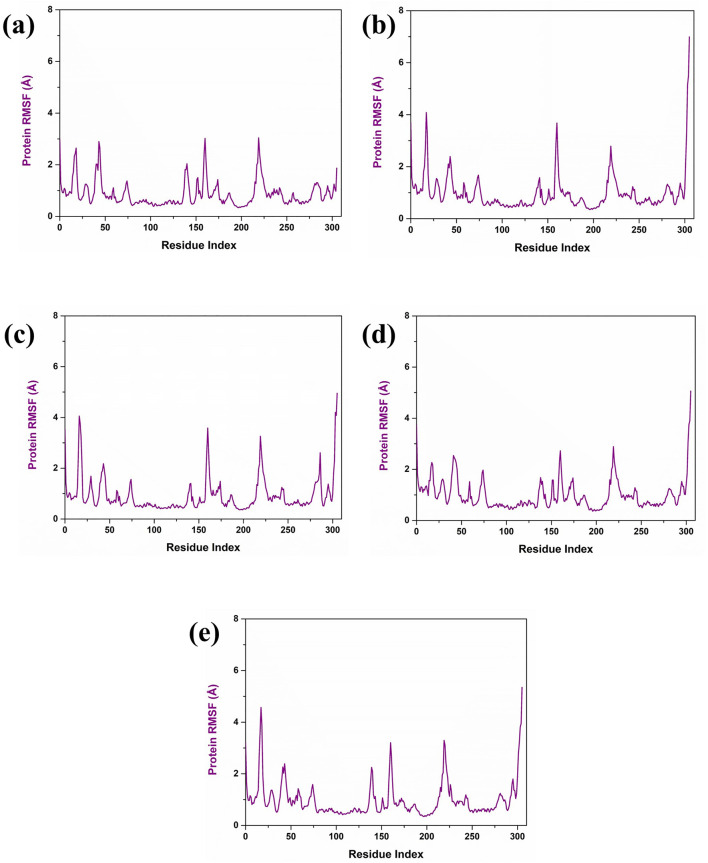
Protein RMSF (violet colour) plot of Yck2-marine complexes viz., **(A)** CMNPD27283, **(B)** CMNPD19660, **(C)** CMNPD27166, and **(D)** CMNPD24402 and **(E)** control (Q0J).

Further, the RMSF analysis of the protein structure was also studied. The RMSF plot analysis of the protein present in each simulated complex showed that the residual regions between 150 and 250 are the prime flexible regions of the protein. Also, the residues in the C terminal end of the protein showed fluctuation, which is considerably negligible. According to the RMSF plot, it was observed that the RMSF value of the protein was less than 4 Å in each complex. As illustrated in Figure 4A, the RMSF value of the protein in the complex CMNPD27283 was 3 Å. In the case of the Yck2-CMNPD19660 and Yck2- CMNPD27166 complexes, the protein RMSF was less than 4 Å (Figures 4B, C). In Yck2-CMNPD24402, the RMSF value was around 2 Å, and for the comparative analysis, it was observed that the control complex also showed the protein RMSF was less than 4 Å (Figures 4D, E). This value indicates that the protein has not undergone conformational changes due to the selected compounds’ binding and remained stable during the simulation. This also suggested that ligands may have the ability to inhibit the function of the protein.

#### 3.3.3 Radius of gyration analysis

**FIGURE 5:**
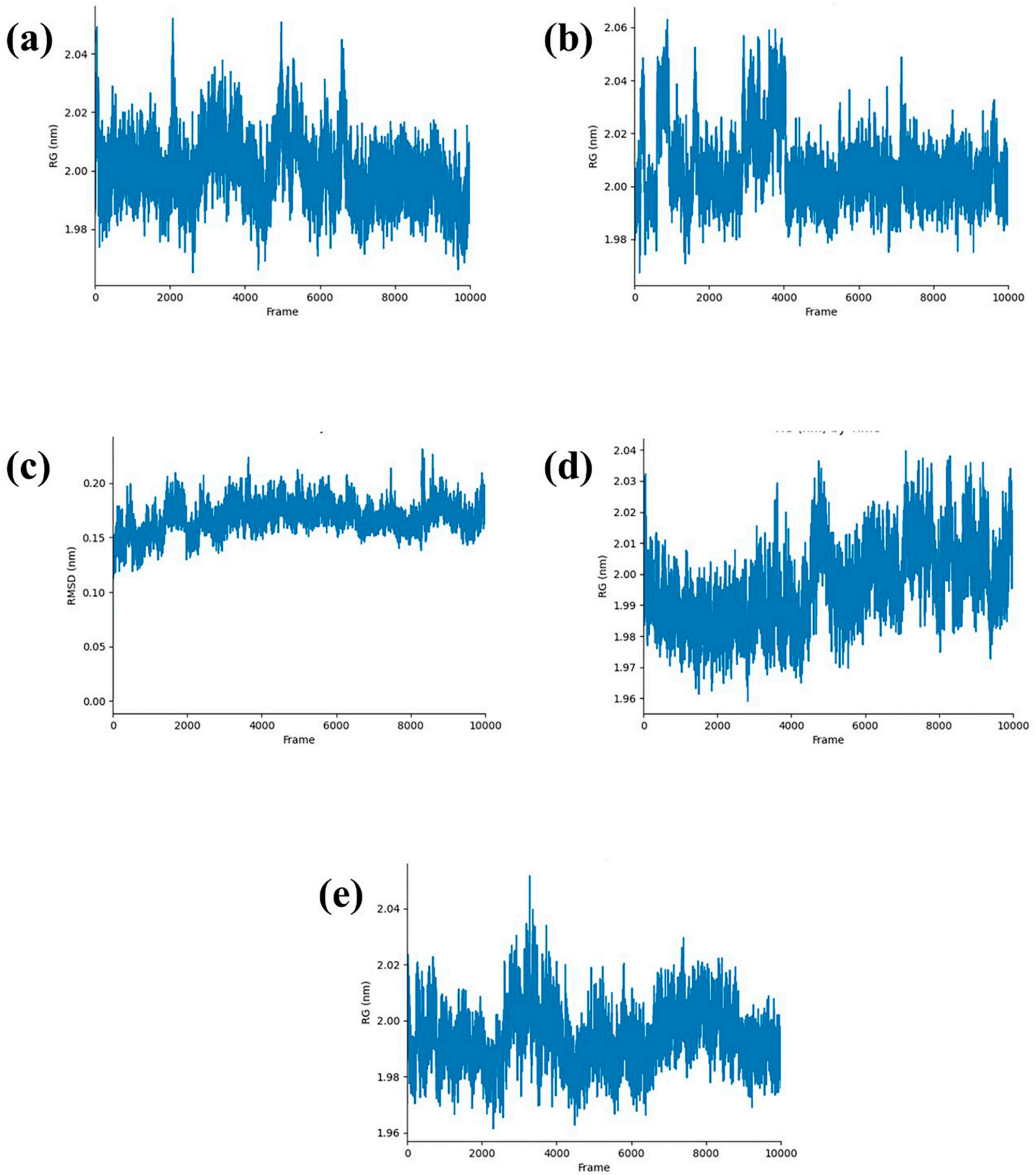
RG plot of Yck2-marine complexes viz., **(A)** CMNPD27283, **(B)** CMNPD19660, **(C)** CMNPD27166, **(D)** CMNPD24402 and **(E)** control (Q0J).

The radius of gyration (Rg) measures how compact a protein’s structure is, which helps us understand its stability. For the Yck2-CMNPD27283 complex, the Rg fluctuated between 2,000 and 6,000 frames, reaching up to 2.04 nm, but stabilized at 2.00 nm after 6,000 frames (Figure 5A). In the Yck2-CMNPD19660 complex, the Rg peaked at 2.06 nm between 0 to 2,000 frames and 3,000 to 4,000 frames, then dropped to 2.00 nm after 4,000 frames (Figure 5B). Throughout the simulation, the Yck2-CMNPD27166 complex showed a stable Rg around 0.15 nm–2.00 nm (Figure 5C). For the Yck2-CMNPD24402 complex, the Rg fluctuated but averaged around 2.04 nm (Figure 5D). The reference complex also had fluctuations, reaching 2.04 nm between 2,000 and 4,000 frames, then stabilizing at 2.04 nm (Figure 5E). These results indicate that all the complexes are quite compact and similar to the reference, with no significant conformational changes.

#### 3.3.4 Hydrogen bond analysis

**FIGURE 6:**
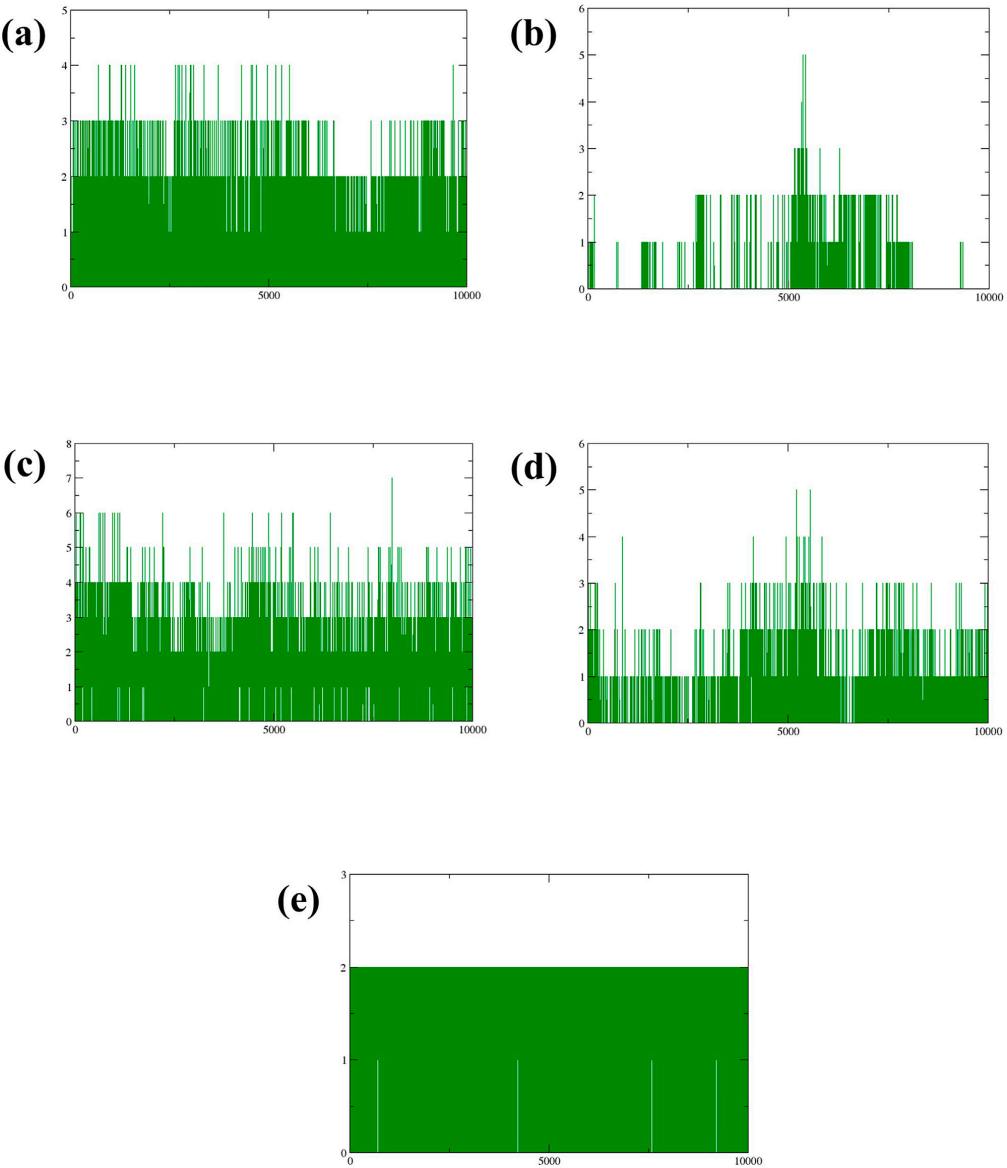
Plot of number of Hydrogen bonds (green colour) formed during the simulation of Yck2-marine complexes viz., **(A)** CMNPD27283, **(B)** CMNPD19660, **(C)** CMNPD27166, and **(D)** CMNPD24402 and **(E)** control (Q0J).

Hydrogen bonds play a crucial role in stabilizing molecular structures, particularly in biological systems (Yunta, 2017). The strength and number of hydrogen bonds can significantly affect molecular conformation and stability, making them a key factor in understanding protein-ligand interactions during simulations. Examining the formation and persistence of hydrogen bonds gave aspects of molecular behavior and potential therapeutic efficacy in drug design. According to Figure 6A, it was observed that during the 300 ns molecular dynamics simulation, compound CMNPD27283 consistently formed two to three hydrogen bonds. In contrast, compound CMNPD19660 generally maintained a single hydrogen bond, though it formed two hydrogen bonds at various points throughout the simulation (Figure 6B). Compound CMNPD27166 exhibited a slightly higher stability in hydrogen bonding, maintaining three to four bonds up to the final frame. At various intervals, 5 to 6 bonds were also formed in this complex (Figure 6C). Compound CMNPD24402 typically formed one to two hydrogen bonds throughout the simulation, but there were intervals where three bonds were observed (Figure 6D). Comparatively, the control compound consistently formed two stable hydrogen bonds till the 300 ns simulation (Figure 6E). These results confirm that the Yck2-CMNPD27166 complex showed maximum stability compared to all other complexes regarding hydrogen bonds.

### 3.4 Binding free energy analysis

The molecular mechanics/generalized Born surface area (MM/GBSA) method was employed to assess the binding free energies of various complexes quantitatively (Genheden and Ryde, 2015). This approach allows for decomposing the total binding energy (ΔG) into its energy components. Such a breakdown facilitates thoroughly examining the energy contributions from interactions between the ligand and the target protein, as outlined in Table 3. The compound CMNPD27283 exhibited the ΔG of −67.12 ± 8.40 kcal/mol. While the compound CMNPD19660 exhibited the ΔG of −50.21 ± 9.67 kcal/mol. The compound CMNPD27166 exhibited the ΔG of −81.67 ± 14.13 kcal/mol. While the compound CMNPD24402 exhibited the ΔG of 47.45 ± 6.28 kcal/mol. On the other hand, the control compound exhibited the ΔG of −47.57 ± 6.77 kcal/mol. The ΔG values show that the studied complexes have strong and stable binding interactions with the target protein, similar to the control complex. Among the energy components, van der Waals energy contributes the most to stability. CMNPD27166 has the highest van der Waals energy (−58.35 ± 2.92 kcal/mol), while CMNPD19660 has the lowest (−39.83 ± 1.98 kcal/mol) (Table 3). The close alignment with the control indicates effective binding dynamics. The small standard deviations suggest high reliability and reproducibility in the energy measurements. These findings show that the compounds have a strong affinity for the target protein, making them effective ligands that can modulate protein activity. This highlights their potential as potent inhibitors in biological pathways.

**TABLE 3:** MMGBSA analysis of four selected compounds in complex with the protein (a) CMNPD27283, (b) CMNPD19660, (c) CMNPD27166, and (d) CMNPD24402 and (e) control (Q0J).

Energy components/Complexes	CMNPD27283	CMNPD19660	CMNPD27166	CMNPD24402	Control
Van der Waal energy (ΔVDWAALS)	−58.35 ± 2.92	−39.83 ± 1.98	−77.09 ± 3.26	−53.43 ± 2.49	−43.24 ± 2.02
Electrostatic energy (ΔEEL)	−23.35 ± 2.14	−8.38 ± 2.54	−23.62 ± 5.12	−17.42 ± 3.78	−7.99 ± 1.58
Polar solvation energy (ΔEGB)	35.39 ± 2.49	19.08 ± 3.11	43.24 ± 4.03	34.71 ± 2.44	24.62 ± 2.15
Non-polar solvation energy (ΔESURF)	−19.81 ± 0.83	−21.09 ± 2.03	−24.18 ± 1.70	−11.31 ± 1.05	−20.95 ± 1.01
Net gas phase energy (ΔGGAS)	−81.70 ± 5.06	−48.21 ± 4.52	−100.72 ± 8.39	−70.85 ± 6.28	−51.24 ± 3.60
Net solvation energy (ΔGSOLV)	14.58 ± 3.33	−2.00 ± 5.15	19.05 ± 5.74	23.40 ± 3.50	3.66 ± 3.17
ΔG_total_	−67.12 ± 8.40	−50.21 ± 9.67	−81.67 ± 14.13	−47.45 ± 6.28	−47.57 ± 6.77

### 3.5 RG-RMSD base free energy analysis

**FIGURE 7:**
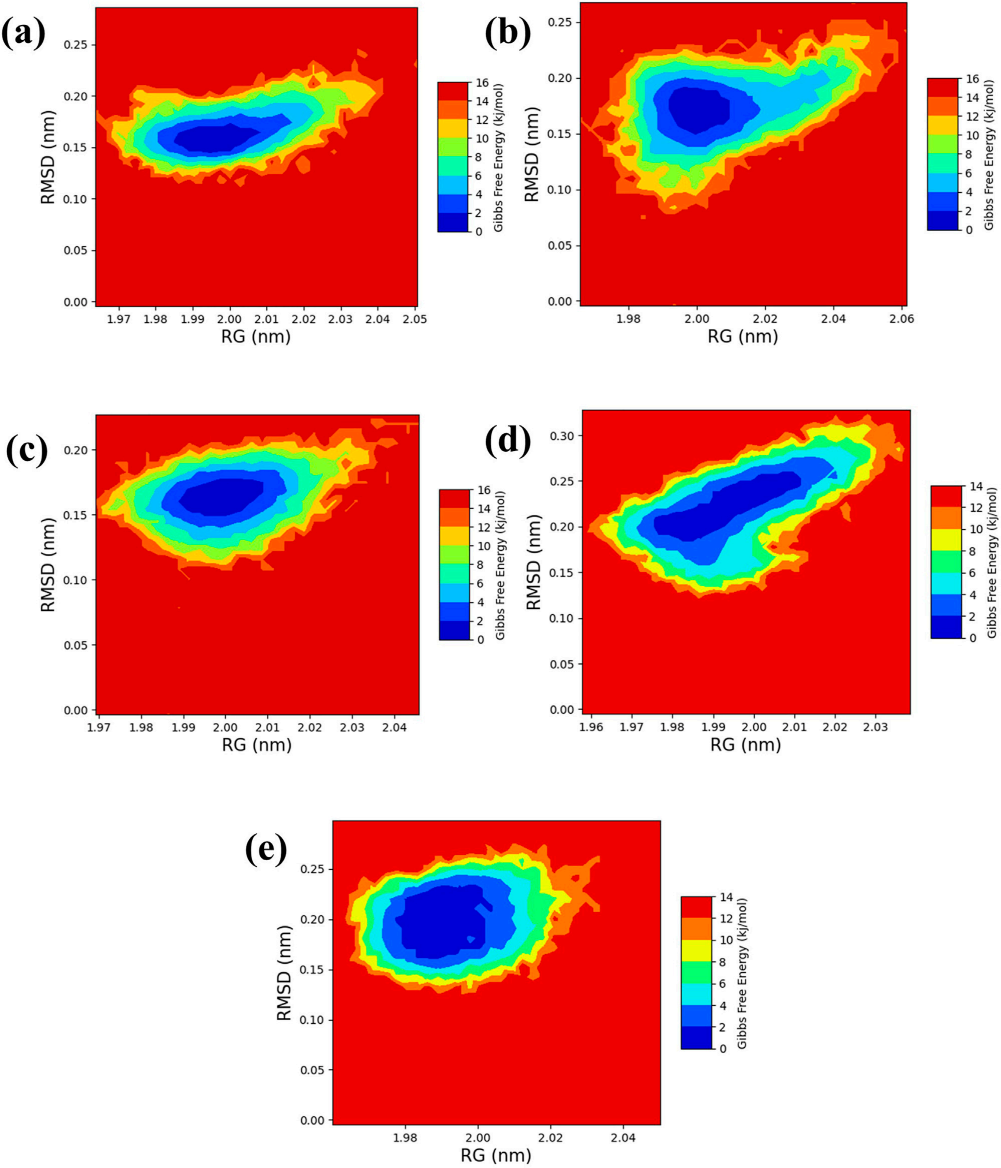
2D RG-RMSD based FEL plot Yck2-marine complexes viz., **(A)** CMNPD27283, **(B)** CMNPD19660, **(C)** CMNPD27166, and **(D)** CMNPD24402 and **(E)** control (Q0J).

**FIGURE 8:**
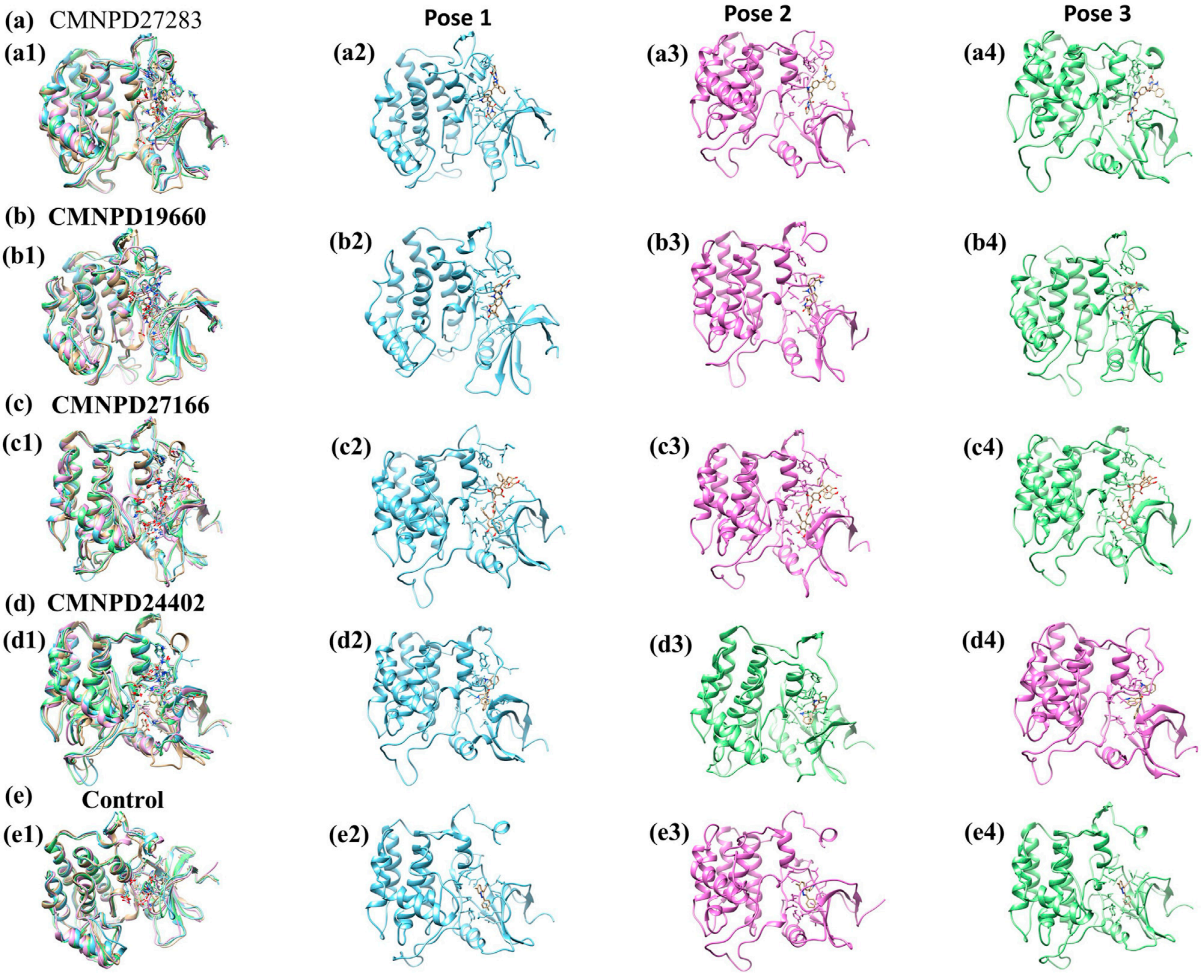
Superimposition of minimum energy poses of Yck2-marine complexes viz., **(A)** CMNPD27283, **(B)** CMNPD19660, **(C)** CMNPD27166, and **(D)** CMNPD24402 and **(E)** control (Q0J) extracted from the simulation trajectory. Herein, the superimposed structures are represented in multicolour. The first minimum energy pose is represented in blue colour, the second pose in pink colour and the third pose in green colour.

The free energy landscape is essential in molecular dynamics simulations, elucidating the energy distribution across biomolecular systems and their conformational dynamics (Bhattacharyya and Vishveshwara, 2010). This landscape is pivotal for comprehending the thermodynamic and kinetic attributes that dictate the behavior of complex biomolecular entities. It delineates energy barriers, stable states, and transitional pathways among molecular conformations. A thorough understanding of thermodynamics and kinetics within this landscape is crucial for analyzing complex interactions, structural transformations, and stability of biomolecular complexes. Additionally, the radius of gyration (RG) analysis offers insights into the protein folding process by indicating the extent of compaction or expansion as the protein folds. Conversely, RMSD analysis provides a measure of the dynamic stability of the complex by tracking deviations from a reference conformation throughout the simulation. To visually represent the 2D and 3D free energy landscapes, graphical depictions were generated using the geo-measure plugin in PyMOL (Kagami et al., 2020), correlating RG with RMSD values. These visualizations aid in interpreting the energy distribution and structural transitions within the biomolecular complex. A methodical framework was implemented to analyze ligand-bound complexes alongside a control group, utilizing graphical representations to enhance understanding of energy dynamics resulting from conformational changes within the studied chemical systems. The two-dimensional (Figure 7 **)** and three-dimensional (Supplementary Figure S1) visualizations effectively demonstrate the dynamic shifts in conformation. This visual methodology aided in identifying a low-energy conformation, revealing a structurally emergent form that provides crucial information about the energetically favorable states of the chemical system. The appearance of a deep blue region within the extensive free energy landscape indicates the presence of localized energy minima, which were consistently observed as the protein structures transitioned into their lowest energy configurations. These minima, marked distinctly by dark blue zones, highlight the organization of chemical groups within minimal energy states. It was repeatedly noted that these complexes maintained localized energy minima within a broader free energy context, as denoted by these dark blue areas. Further examination of the free energy landscape in two and three dimensions reveals the thermodynamic properties of the chemical complexes studied, including a control compound. These analyses provide comprehensive insights into their molecular dynamics and stability profiles. A notable feature of this landscape is the consistent presence of a relative maximal energy state among these complexes, sustained within an energy range of 14–16 kJ/mol. This consistency across the complexes suggests a potential homogeneity in structural or functional attributes, such as molecular interactions or stability. Additionally, all complexes exhibited a stable conformation at energy levels below 2 kJ/mol, as depicted by the dark blue area on the landscape, confirming their thermodynamic stability. It focused on identifying lower energy states for each complex and subsequently extracted three particularly stable poses from them. To evaluate these poses’ structural alignment and consistency, the superimposed structures were generated by aligning each of the three stable poses with the initial pose, which served as the reference structure, as illustrated in Figure 8. This alignment using the RMSD was quantitatively assessed, a standard structural biology metric measuring the average distance between atoms of superimposed structures after optimal alignment. A lower RMSD value indicates higher structural similarity or congruence between compared structures.

The RMSD values indicate the structural integrity and consistency across the superimposed poses, reflecting their stability. Specifically, compound CMNPD27283 exhibited an RMSD of 1.288 Å, suggesting a close alignment with the reference pose and robust consistency across the stable poses. Compound CMNPD19660 showed an RMSD of 1.382 Å, and compound CMNPD27166 had an RMSD of 1.304 Å, demonstrating good structural alignment. Compound CMNPD24402s RMSD was 1.381 Å, which, like CMNPD19660, reflects good structural consistency. Comparatively, the control compound’s superimposed structure exhibited an RMSD of 1.297 Å, showing a high degree of alignment comparable to that of the experimental compounds. These RMSD values provide valuable insights into the structural fidelity of the superimposed poses relative to the initial pose, confirming that the extracted stable poses represent each complex’s structural characteristics in their low-energy conformations. Such detailed analysis is essential for understanding the dynamic structural behavior of biomolecular complexes, especially in MD simulations where the stability and variability of molecular conformations can significantly impact biological function and interactions.

## 4 Discussion

*C*. *albicans* is an opportunistic fungal pathogen responsible for infections linked to high mortality. The previous study has shown that *C. albicans* Yeast Casein Kinase 2 (CaYck2) regulates the yeast-to-hyphal switch, and this transition from yeast to hyphae plays a major role in the virulence of C. albicans. It presents a promising focus for the development of antifungal medications. Yeast casein kinase (Yck2) plays a crucial role in cell wall regulation and has been identified as a promising drug target for combating drug resistance in *C. albicans.* Our study is based on a comprehensive computational approach to identify and characterize potential inhibitors of Yck2 from *C. albicans* derived from marine bacterial compounds. Computational drug discovery approaches have been a significant contributor to the identification of potential inhibitors of CaYck2. Various research groups have predominately used this method of identification. Recently, Hassan and his team identified five compounds from the PubChem database that are structurally similar analogs of previously identified kinase inhibitors (Hassan et al., 2024). Similarly, Rabaan and his research team also targeted the Yck2 protein and identified five drug-like compounds using computational drug discovery approaches. These studies confirmed that computational approaches have been the leading method for inhibitor identification (Rabaan et al., 2023). Marine compounds are one of the important sources for finding drug-like compounds. In this experiment, I have virtually screened around 2,895 marine bacteria, and from these five marine compounds, Naseseazine C (CMNPD27283), Dermacozine E (CMNPD19660), Wailupemycin H (CMNPD27166), and metagenetriindole A (CMNPD24402). Naseseazine C is a diketopiperazine isolated from Australian marine actinomycete. This compound exhibited moderate anti-plasmodial and anti-malaria parasite activity (Buedenbender et al., 2016). Also, a recent study found this compound as a potential inhibitor of the hepatitis C virus polymerase (Alobaida et al., 2024). This compound showed a binding energy of −12.8 kcal/mol during the screening against the Yck2 protein. Likewise, the Dermacozine E, a phenazine derivative obtained from the actinomycete Dermacoccus abyssi sp., showed cytotoxic activity against cancer cell lines. Also, this compound exhibited radial scavenging activity and antioxidant properties (Abdel-Mageed et al., 2010). Furthermore, the compound Wailupemycin H is an epimeric polyketide extracted from the fermentation broth of *Streptomyces* sp. OUCMDZ-3434 combined with marine green algae, *Enteromorpha prolifera*, which exhibited promising α-glucosidase inhibitory activity, can be used to treat diabetes mellitus (Chen et al., 2016). Metagenetriindole A is a bisindole alkaloid isolated from deep-sea sediment metagenomic clone-derived *Escherichia coli* fermentation broth. They showed cytotoxic, antiplasmodial, and antimicrobial properties (Pon Sathieshkumar and Nagarajan, 2017).

The molecular interaction analysis of these compounds, when docked with Yck2 protein, showed a significant amount of hydrophobic bond formation along with hydrogen bonds. These bonds contributed to developing maximum binding stability. A similar scenario was also observed in the research work conducted by Hassan and the team (Hasan et al., 2022).

CMNPD27283 displays a more favorable ADMET profile, making it a more promising candidate for therapeutic use. With a molecular weight of 564.63 g/mol and only one Lipinski violation, CMNPD27283 demonstrates reasonable drug-likeness. It is classified as soluble and exhibits good gastrointestinal absorption, suggesting it could be effectively administered orally. Furthermore, it shows moderate toxicity and is not a blood-brain barrier permeant, which can reduce potential central nervous system side effects. In contrast, CMNPD27166 has a higher molecular weight (722.69 g/mol) and three Lipinski violations, indicating poorer drug-like properties. Its low solubility and gastrointestinal absorption further limit its potential for oral delivery. Despite these drawbacks, it is also a P-glycoprotein substrate and displays moderate toxicity without raising significant toxicity alerts. While CMNPD27283 appears to have more desirable pharmacokinetic characteristics, CMNPD27166 may require additional optimization to overcome its limitations.

Furthermore, the molecular dynamics simulation trajectory analysis also proved the stability of these compounds by calculating the RMSD, RMSF, and the hydrogen binding pattern analysis. The RMSD analysis showed that CMNPD27166 showed maximum dynamic stability. Similar conditions were observed in the previously identified inhibitors (PubChem IDs 102583821, 12982634, 102487860, and 86260205) (Hasan et al., 2022). The RMSF analysis of the protein structure proved that the binding of these selected marine compounds does not lead to structural conformational changes. Interestingly, the residual fluctuations were less in the protein structure than those bounded by the previously identified structural analog and drug-like compound inhibitors (Rabaan et al., 2024; Hassan et al., 2024). After the simulation, the hydrogen bond formation analysis also confirmed that all the compounds formed at least two hydrogens, and a maximum of four hydrogen bonds were displayed in the CMNPD27166 compound. Also, the free binding energy and free energy landscape analysis supported these findings. The comparison of these findings with previous studies performed by Hassan and his co-workers in 2024 suggested that these marine compounds also showed similar inhibitory properties to the previously identified inhibitors (Hassan et al., 2024). This also concludes that these marine compounds exhibit inhibitory activity like the previously identified inhibitors against the Yck2 protein and can be a significant part of anti-fungal drugs.

## 5 Conclusion

This study demonstrated the effective use of a multi-dimensional computational drug discovery approach to identify and evaluate potential inhibitors of Yck2, a key regulatory protein in *C. albicans*. Through meticulous virtual screening, re-docking, molecular dynamics simulations, and various analytical methods, this study identified four promising compounds (CMNPD27283, CMNPD19660, CMNPD27166, and CMNPD24402) from the marine bacterial compound database, which exhibited potential as Yck2 inhibitors. CMNPD27166 and CMNPD27283 emerged as the most promising candidates, showing superior binding affinity, stability, and favorable interaction dynamics with the Yck2 protein. These compounds consistently outperformed the control in multiple metrics, including docking scores, hydrogen bonding, RMSD stability, and free-binding energy calculations. Their lower energy states in RG-RMSD-based free energy landscapes corroborate their potential efficacy and stability as inhibitors. CMNPD19660 and CMNPD24402, while effective to a degree, demonstrated less optimal profiles than the control and other compounds. Their higher flexibility and less favorable binding energies suggest that while they possess inhibitory capabilities, their therapeutic efficacy might be lower. Our study’s findings highlight the capabilities of computational techniques in rapidly screening and evaluating bioactive compounds and illuminate the therapeutic potential of marine-derived molecules in the treatment of diseases dependent on the modulation of Yck2. Furthermore, this research underscores the importance of utilizing comprehensive computational approaches to pre-screen and refine candidates before advancing to experimental validation, thus enhancing the efficiency and efficacy of drug discovery processes. The compounds CMNPD27166 and CMNPD27283 are recommended for further experimental studies to confirm their potential as effective antifungal agents against echinocandin-resistant *C. albicans* infections.

## Data Availability

The original contributions presented in the study are included in the article/Supplementary Material, further inquiries can be directed to the corresponding author.
