# Supplementary material for: Antifungal potential of marine bacterial compounds in inhibiting Candida albicans Yck2 to overcome echinocandin resistance: a molecular dynamics study
Source: Front Pharmacol. 2024 Oct 17;15:1459964. doi: 10.3389/fphar.2024.1459964 (PMC11525067; doi:10.3389/fphar.2024.1459964)
Supplement: Supplementary file 1 [file Table1.DOCX]

Supplementary Material

**Table S1**- List of 1500 selected compound after the virtual screening with their energy.

| **S.no** | **Compound** | **Energy** |
| --- | --- | --- |
| 1 | CMNPD27283 | -12.7 |
| 2 | CMNPD19660 | -12.2 |
| 3 | CMNPD27166 | -12 |
| 4 | CMNPD24402 | -12 |
| 5 | CMNPD18676 | -11.9 |
| 6 | CMNPD18675 | -11.9 |
| 7 | CMNPD30129 | -11.6 |
| 8 | CMNPD19661 | -11.6 |
| 9 | CMNPD5875 | -11.5 |
| 10 | CMNPD21065 | -11.5 |
| 11 | CMNPD25830 | -11.4 |
| 12 | CMNPD30131 | -11.4 |
| 13 | CMNPD30130 | -11.4 |
| 14 | CMNPD20698 | -11.3 |
| 15 | CMNPD28613 | -11.3 |
| 16 | CMNPD24471 | -11.3 |
| 17 | CMNPD16561 | -11.2 |
| 18 | CMNPD27136 | -11.1 |
| 19 | CMNPD18673 | -11.1 |
| 20 | CMNPD28413 | -11.1 |
| 21 | CMNPD21926 | -11 |
| 22 | CMNPD9392 | -11 |
| 23 | CMNPD15675 | -11 |
| 24 | CMNPD25797 | -10.9 |
| 25 | CMNPD14710 | -10.9 |
| 26 | CMNPD17537 | -10.8 |
| 27 | CMNPD13211 | -10.8 |
| 28 | CMNPD30014 | -10.8 |
| 29 | CMNPD30142 | -10.7 |
| 30 | CMNPD16562 | -10.7 |
| 31 | CMNPD20696 | -10.7 |
| 32 | CMNPD9391 | -10.7 |
| 33 | CMNPD25800 | -10.7 |
| 34 | CMNPD21066 | -10.6 |
| 35 | CMNPD24355 | -10.6 |
| 36 | CMNPD30029 | -10.6 |
| 37 | CMNPD9394 | -10.6 |
| 38 | CMNPD21973 | -10.6 |
| 39 | CMNPD13253 | -10.6 |
| 40 | CMNPD28406 | -10.6 |
| 41 | CMNPD28412 | -10.6 |
| 42 | CMNPD28415 | -10.6 |
| 43 | CMNPD15676 | -10.5 |
| 44 | CMNPD21948 | -10.5 |
| 45 | CMNPD30018 | -10.5 |
| 46 | CMNPD25807 | -10.5 |
| 47 | CMNPD19662 | -10.5 |
| 48 | CMNPD24354 | -10.5 |
| 49 | CMNPD30016 | -10.5 |
| 50 | CMNPD30020 | -10.4 |
| 51 | CMNPD28642 | -10.4 |
| 52 | CMNPD20697 | -10.4 |
| 53 | CMNPD19681 | -10.4 |
| 54 | CMNPD13187 | -10.4 |
| 55 | CMNPD30030 | -10.4 |
| 56 | CMNPD26391 | -10.4 |
| 57 | CMNPD19702 | -10.4 |
| 58 | CMNPD23224 | -10.4 |
| 59 | CMNPD10900 | -10.3 |
| 60 | CMNPD25806 | -10.3 |
| 61 | CMNPD30027 | -10.3 |
| 62 | CMNPD19658 | -10.3 |
| 63 | CMNPD27176 | -10.3 |
| 64 | CMNPD30068 | -10.3 |
| 65 | CMNPD20714 | -10.3 |
| 66 | CMNPD30019 | -10.3 |
| 67 | CMNPD27247 | -10.3 |
| 68 | CMNPD19680 | -10.3 |
| 69 | CMNPD16733 | -10.3 |
| 70 | CMNPD20715 | -10.3 |
| 71 | CMNPD25801 | -10.2 |
| 72 | CMNPD23169 | -10.2 |
| 73 | CMNPD1749 | -10.2 |
| 74 | CMNPD19653 | -10.2 |
| 75 | CMNPD30067 | -10.2 |
| 76 | CMNPD30148 | -10.2 |
| 77 | CMNPD30138 | -10.2 |
| 78 | CMNPD23211 | -10.2 |
| 79 | CMNPD10129 | -10.2 |
| 80 | CMNPD241 | -10.2 |
| 81 | CMNPD23265 | -10.2 |
| 82 | CMNPD17535 | -10.2 |
| 83 | CMNPD21963 | -10.2 |
| 84 | CMNPD21902 | -10.2 |
| 85 | CMNPD9393 | -10.1 |
| 86 | CMNPD7236 | -10.1 |
| 87 | CMNPD30017 | -10.1 |
| 88 | CMNPD28646 | -10.1 |
| 89 | CMNPD24408 | -10.1 |
| 90 | CMNPD19699 | -10.1 |
| 91 | CMNPD17540 | -10.1 |
| 92 | CMNPD30036 | -10.1 |
| 93 | CMNPD30143 | -10.1 |
| 94 | CMNPD21970 | -10.1 |
| 95 | CMNPD19682 | -10.1 |
| 96 | CMNPD30135 | -10 |
| 97 | CMNPD25880 | -10 |
| 98 | CMNPD30002 | -10 |
| 99 | CMNPD19896 | -10 |
| 100 | CMNPD21938 | -10 |
| 101 | CMNPD24409 | -10 |
| 102 | CMNPD27275 | -10 |
| 103 | CMNPD28414 | -10 |
| 104 | CMNPD23213 | -10 |
| 105 | CMNPD20739 | -10 |
| 106 | CMNPD19696 | -10 |
| 107 | CMNPD19647 | -10 |
| 108 | CMNPD2197 | -10 |
| 109 | CMNPD17568 | -10 |
| 110 | CMNPD27163 | -10 |
| 111 | CMNPD27111 | -10 |
| 112 | CMNPD23301 | -10 |
| 113 | CMNPD30031 | -10 |
| 114 | CMNPD6531 | -10 |
| 115 | CMNPD335 | -10 |
| 116 | CMNPD23150 | -10 |
| 117 | CMNPD20740 | -10 |
| 118 | CMNPD19652 | -10 |
| 119 | CMNPD6530 | -10 |
| 120 | CMNPD23311 | -10 |
| 121 | CMNPD25866 | -10 |
| 122 | CMNPD19729 | -9.9 |
| 123 | CMNPD27134 | -9.9 |
| 124 | CMNPD19657 | -9.9 |
| 125 | CMNPD27216 | -9.9 |
| 126 | CMNPD24376 | -9.9 |
| 127 | CMNPD28488 | -9.9 |
| 128 | CMNPD25779 | -9.9 |
| 129 | CMNPD23225 | -9.9 |
| 130 | CMNPD17538 | -9.9 |
| 131 | CMNPD17563 | -9.9 |
| 132 | CMNPD6532 | -9.9 |
| 133 | CMNPD25746 | -9.9 |
| 134 | CMNPD21979 | -9.9 |
| 135 | CMNPD19691 | -9.9 |
| 136 | CMNPD23207 | -9.9 |
| 137 | CMNPD20782 | -9.9 |
| 138 | CMNPD23216 | -9.9 |
| 139 | CMNPD24369 | -9.9 |
| 140 | CMNPD5306 | -9.8 |
| 141 | CMNPD24400 | -9.8 |
| 142 | CMNPD17558 | -9.8 |
| 143 | CMNPD30149 | -9.8 |
| 144 | CMNPD14685 | -9.8 |
| 145 | CMNPD27170 | -9.8 |
| 146 | CMNPD27178 | -9.8 |
| 147 | CMNPD30134 | -9.8 |
| 148 | CMNPD6529 | -9.8 |
| 149 | CMNPD27140 | -9.8 |
| 150 | CMNPD19677 | -9.8 |
| 151 | CMNPD18628 | -9.8 |
| 152 | CMNPD19897 | -9.8 |
| 153 | CMNPD6536 | -9.8 |
| 154 | CMNPD23263 | -9.8 |
| 155 | CMNPD30035 | -9.8 |
| 156 | CMNPD23168 | -9.8 |
| 157 | CMNPD20768 | -9.8 |
| 158 | CMNPD17536 | -9.8 |
| 159 | CMNPD19740 | -9.8 |
| 160 | CMNPD20709 | -9.7 |
| 161 | CMNPD25796 | -9.7 |
| 162 | CMNPD23217 | -9.7 |
| 163 | CMNPD27135 | -9.7 |
| 164 | CMNPD30024 | -9.7 |
| 165 | CMNPD19739 | -9.7 |
| 166 | CMNPD24482 | -9.7 |
| 167 | CMNPD23256 | -9.7 |
| 168 | CMNPD25802 | -9.7 |
| 169 | CMNPD30153 | -9.7 |
| 170 | CMNPD27164 | -9.7 |
| 171 | CMNPD19678 | -9.7 |
| 172 | CMNPD27169 | -9.7 |
| 173 | CMNPD10130 | -9.7 |
| 174 | CMNPD21924 | -9.7 |
| 175 | CMNPD12485 | -9.7 |
| 176 | CMNPD20744 | -9.7 |
| 177 | CMNPD28501 | -9.7 |
| 178 | CMNPD14706 | -9.7 |
| 179 | CMNPD28451 | -9.7 |
| 180 | CMNPD18667 | -9.7 |
| 181 | CMNPD20741 | -9.7 |
| 182 | CMNPD10901 | -9.7 |
| 183 | CMNPD19692 | -9.7 |
| 184 | CMNPD27165 | -9.7 |
| 185 | CMNPD21903 | -9.6 |
| 186 | CMNPD334 | -9.6 |
| 187 | CMNPD21968 | -9.6 |
| 188 | CMNPD18679 | -9.6 |
| 189 | CMNPD28561 | -9.6 |
| 190 | CMNPD25939 | -9.6 |
| 191 | CMNPD18665 | -9.6 |
| 192 | CMNPD13300 | -9.6 |
| 193 | CMNPD30136 | -9.6 |
| 194 | CMNPD19703 | -9.6 |
| 195 | CMNPD25725 | -9.6 |
| 196 | CMNPD23228 | -9.6 |
| 197 | CMNPD28647 | -9.6 |
| 198 | CMNPD30145 | -9.6 |
| 199 | CMNPD24449 | -9.6 |
| 200 | CMNPD24971 | -9.6 |
| 201 | CMNPD18650 | -9.6 |
| 202 | CMNPD24483 | -9.6 |
| 203 | CMNPD23221 | -9.6 |
| 204 | CMNPD30150 | -9.6 |
| 205 | CMNPD339 | -9.6 |
| 206 | CMNPD19679 | -9.6 |
| 207 | CMNPD30133 | -9.5 |
| 208 | CMNPD17567 | -9.5 |
| 209 | CMNPD2189 | -9.5 |
| 210 | CMNPD24450 | -9.5 |
| 211 | CMNPD28550 | -9.5 |
| 212 | CMNPD7284 | -9.5 |
| 213 | CMNPD30217 | -9.5 |
| 214 | CMNPD28500 | -9.5 |
| 215 | CMNPD19697 | -9.5 |
| 216 | CMNPD18666 | -9.5 |
| 217 | CMNPD30028 | -9.5 |
| 218 | CMNPD28570 | -9.5 |
| 219 | CMNPD30126 | -9.5 |
| 220 | CMNPD18660 | -9.5 |
| 221 | CMNPD28470 | -9.5 |
| 222 | CMNPD13886 | -9.5 |
| 223 | CMNPD17586 | -9.5 |
| 224 | CMNPD27137 | -9.5 |
| 225 | CMNPD30144 | -9.5 |
| 226 | CMNPD28638 | -9.5 |
| 227 | CMNPD23289 | -9.5 |
| 228 | CMNPD27138 | -9.5 |
| 229 | CMNPD21941 | -9.5 |
| 230 | CMNPD21901 | -9.5 |
| 231 | CMNPD20695 | -9.5 |
| 232 | CMNPD29996 | -9.4 |
| 233 | CMNPD25778 | -9.4 |
| 234 | CMNPD28578 | -9.4 |
| 235 | CMNPD28571 | -9.4 |
| 236 | CMNPD25817 | -9.4 |
| 237 | CMNPD23212 | -9.4 |
| 238 | CMNPD27228 | -9.4 |
| 239 | CMNPD24397 | -9.4 |
| 240 | CMNPD341 | -9.4 |
| 241 | CMNPD25859 | -9.4 |
| 242 | CMNPD17564 | -9.4 |
| 243 | CMNPD30048 | -9.4 |
| 244 | CMNPD13212 | -9.4 |
| 245 | CMNPD25788 | -9.4 |
| 246 | CMNPD24481 | -9.4 |
| 247 | CMNPD21895 | -9.4 |
| 248 | CMNPD21899 | -9.4 |
| 249 | CMNPD25729 | -9.4 |
| 250 | CMNPD21914 | -9.4 |
| 251 | CMNPD23219 | -9.4 |
| 252 | CMNPD5309 | -9.4 |
| 253 | CMNPD18648 | -9.4 |
| 254 | CMNPD28536 | -9.4 |
| 255 | CMNPD28563 | -9.4 |
| 256 | CMNPD14698 | -9.4 |
| 257 | CMNPD21900 | -9.4 |
| 258 | CMNPD17534 | -9.4 |
| 259 | CMNPD14709 | -9.3 |
| 260 | CMNPD21896 | -9.3 |
| 261 | CMNPD19656 | -9.3 |
| 262 | CMNPD28480 | -9.3 |
| 263 | CMNPD17541 | -9.3 |
| 264 | CMNPD23220 | -9.3 |
| 265 | CMNPD7281 | -9.3 |
| 266 | CMNPD30140 | -9.3 |
| 267 | CMNPD19690 | -9.3 |
| 268 | CMNPD24484 | -9.3 |
| 269 | CMNPD16546 | -9.3 |
| 270 | CMNPD13192 | -9.3 |
| 271 | CMNPD25799 | -9.3 |
| 272 | CMNPD16552 | -9.3 |
| 273 | CMNPD21911 | -9.3 |
| 274 | CMNPD18669 | -9.3 |
| 275 | CMNPD30114 | -9.3 |
| 276 | CMNPD8741 | -9.3 |
| 277 | CMNPD30015 | -9.3 |
| 278 | CMNPD14708 | -9.3 |
| 279 | CMNPD28577 | -9.3 |
| 280 | CMNPD30041 | -9.3 |
| 281 | CMNPD25798 | -9.3 |
| 282 | CMNPD27167 | -9.3 |
| 283 | CMNPD23218 | -9.3 |
| 284 | CMNPD24384 | -9.3 |
| 285 | CMNPD18649 | -9.3 |
| 286 | CMNPD20746 | -9.3 |
| 287 | CMNPD7996 | -9.3 |
| 288 | CMNPD20748 | -9.3 |
| 289 | CMNPD10899 | -9.3 |
| 290 | CMNPD19695 | -9.3 |
| 291 | CMNPD5305 | -9.3 |
| 292 | CMNPD30032 | -9.3 |
| 293 | CMNPD28560 | -9.3 |
| 294 | CMNPD25786 | -9.2 |
| 295 | CMNPD25767 | -9.2 |
| 296 | CMNPD18624 | -9.2 |
| 297 | CMNPD24970 | -9.2 |
| 298 | CMNPD30044 | -9.2 |
| 299 | CMNPD24328 | -9.2 |
| 300 | CMNPD28405 | -9.2 |
| 301 | CMNPD24448 | -9.2 |
| 302 | CMNPD30039 | -9.2 |
| 303 | CMNPD24460 | -9.2 |
| 304 | CMNPD19648 | -9.2 |
| 305 | CMNPD19954 | -9.2 |
| 306 | CMNPD25740 | -9.2 |
| 307 | CMNPD30080 | -9.2 |
| 308 | CMNPD23226 | -9.2 |
| 309 | CMNPD23283 | -9.2 |
| 310 | CMNPD28604 | -9.2 |
| 311 | CMNPD24969 | -9.2 |
| 312 | CMNPD21910 | -9.2 |
| 313 | CMNPD13213 | -9.2 |
| 314 | CMNPD23253 | -9.2 |
| 315 | CMNPD28511 | -9.2 |
| 316 | CMNPD28487 | -9.2 |
| 317 | CMNPD23239 | -9.2 |
| 318 | CMNPD21971 | -9.2 |
| 319 | CMNPD17532 | -9.2 |
| 320 | CMNPD25803 | -9.2 |
| 321 | CMNPD24394 | -9.2 |
| 322 | CMNPD30146 | -9.2 |
| 323 | CMNPD20720 | -9.2 |
| 324 | CMNPD27192 | -9.2 |
| 325 | CMNPD24479 | -9.2 |
| 326 | CMNPD30042 | -9.2 |
| 327 | CMNPD21889 | -9.2 |
| 328 | CMNPD24381 | -9.2 |
| 329 | CMNPD28513 | -9.2 |
| 330 | CMNPD20783 | -9.2 |
| 331 | CMNPD30037 | -9.2 |
| 332 | CMNPD21943 | -9.2 |
| 333 | CMNPD25766 | -9.1 |
| 334 | CMNPD6534 | -9.1 |
| 335 | CMNPD19734 | -9.1 |
| 336 | CMNPD21894 | -9.1 |
| 337 | CMNPD24401 | -9.1 |
| 338 | CMNPD25768 | -9.1 |
| 339 | CMNPD27118 | -9.1 |
| 340 | CMNPD21942 | -9.1 |
| 341 | CMNPD19659 | -9.1 |
| 342 | CMNPD30049 | -9.1 |
| 343 | CMNPD7283 | -9.1 |
| 344 | CMNPD30141 | -9.1 |
| 345 | CMNPD30167 | -9.1 |
| 346 | CMNPD30033 | -9.1 |
| 347 | CMNPD8742 | -9.1 |
| 348 | CMNPD6537 | -9.1 |
| 349 | CMNPD23232 | -9.1 |
| 350 | CMNPD5335 | -9.1 |
| 351 | CMNPD30100 | -9.1 |
| 352 | CMNPD874 | -9.1 |
| 353 | CMNPD28533 | -9.1 |
| 354 | CMNPD20722 | -9.1 |
| 355 | CMNPD23287 | -9.1 |
| 356 | CMNPD5310 | -9.1 |
| 357 | CMNPD24395 | -9.1 |
| 358 | CMNPD1764 | -9.1 |
| 359 | CMNPD5307 | -9.1 |
| 360 | CMNPD10189 | -9.1 |
| 361 | CMNPD240 | -9.1 |
| 362 | CMNPD29995 | -9.1 |
| 363 | CMNPD28510 | -9.1 |
| 364 | CMNPD310 | -9.1 |
| 365 | CMNPD27277 | -9.1 |
| 366 | CMNPD24497 | -9.1 |
| 367 | CMNPD19676 | -9.1 |
| 368 | CMNPD25795 | -9.1 |
| 369 | CMNPD30139 | -9.1 |
| 370 | CMNPD24398 | -9.1 |
| 371 | CMNPD30022 | -9.1 |
| 372 | CMNPD24524 | -9.1 |
| 373 | CMNPD8747 | -9.1 |
| 374 | CMNPD30040 | -9 |
| 375 | CMNPD2571 | -9 |
| 376 | CMNPD30103 | -9 |
| 377 | CMNPD17573 | -9 |
| 378 | CMNPD21904 | -9 |
| 379 | CMNPD21925 | -9 |
| 380 | CMNPD20738 | -9 |
| 381 | CMNPD23242 | -9 |
| 382 | CMNPD19726 | -9 |
| 383 | CMNPD27774 | -9 |
| 384 | CMNPD14683 | -9 |
| 385 | CMNPD15700 | -9 |
| 386 | CMNPD22322 | -9 |
| 387 | CMNPD23294 | -9 |
| 388 | CMNPD20758 | -9 |
| 389 | CMNPD19675 | -9 |
| 390 | CMNPD21933 | -9 |
| 391 | CMNPD21964 | -9 |
| 392 | CMNPD30113 | -9 |
| 393 | CMNPD25749 | -9 |
| 394 | CMNPD20679 | -9 |
| 395 | CMNPD21949 | -9 |
| 396 | CMNPD7280 | -9 |
| 397 | CMNPD19895 | -9 |
| 398 | CMNPD7986 | -9 |
| 399 | CMNPD23208 | -9 |
| 400 | CMNPD1762 | -9 |
| 401 | CMNPD10898 | -9 |
| 402 | CMNPD30026 | -9 |
| 403 | CMNPD13184 | -9 |
| 404 | CMNPD24348 | -9 |
| 405 | CMNPD23161 | -9 |
| 406 | CMNPD15679 | -9 |
| 407 | CMNPD30046 | -9 |
| 408 | CMNPD30043 | -9 |
| 409 | CMNPD27245 | -9 |
| 410 | CMNPD21976 | -9 |
| 411 | CMNPD19725 | -9 |
| 412 | CMNPD27145 | -9 |
| 413 | CMNPD19649 | -9 |
| 414 | CMNPD25789 | -9 |
| 415 | CMNPD5308 | -9 |
| 416 | CMNPD30079 | -9 |
| 417 | CMNPD8749 | -9 |
| 418 | CMNPD4669 | -8.9 |
| 419 | CMNPD18680 | -8.9 |
| 420 | CMNPD24366 | -8.9 |
| 421 | CMNPD21983 | -8.9 |
| 422 | CMNPD20708 | -8.9 |
| 423 | CMNPD23148 | -8.9 |
| 424 | CMNPD17549 | -8.9 |
| 425 | CMNPD7989 | -8.9 |
| 426 | CMNPD25781 | -8.9 |
| 427 | CMNPD342 | -8.9 |
| 428 | CMNPD21937 | -8.9 |
| 429 | CMNPD21959 | -8.9 |
| 430 | CMNPD24367 | -8.9 |
| 431 | CMNPD23147 | -8.9 |
| 432 | CMNPD13214 | -8.9 |
| 433 | CMNPD25785 | -8.9 |
| 434 | CMNPD28452 | -8.9 |
| 435 | CMNPD17545 | -8.9 |
| 436 | CMNPD20681 | -8.9 |
| 437 | CMNPD17539 | -8.9 |
| 438 | CMNPD24403 | -8.9 |
| 439 | CMNPD25868 | -8.9 |
| 440 | CMNPD17531 | -8.9 |
| 441 | CMNPD18664 | -8.9 |
| 442 | CMNPD24466 | -8.9 |
| 443 | CMNPD19718 | -8.9 |
| 444 | CMNPD20719 | -8.9 |
| 445 | CMNPD24350 | -8.9 |
| 446 | CMNPD25884 | -8.9 |
| 447 | CMNPD28607 | -8.9 |
| 448 | CMNPD30052 | -8.9 |
| 449 | CMNPD12381 | -8.9 |
| 450 | CMNPD25870 | -8.9 |
| 451 | CMNPD24459 | -8.9 |
| 452 | CMNPD28475 | -8.9 |
| 453 | CMNPD5311 | -8.9 |
| 454 | CMNPD19712 | -8.9 |
| 455 | CMNPD28450 | -8.9 |
| 456 | CMNPD20759 | -8.9 |
| 457 | CMNPD20868 | -8.9 |
| 458 | CMNPD30021 | -8.9 |
| 459 | CMNPD28411 | -8.9 |
| 460 | CMNPD7990 | -8.9 |
| 461 | CMNPD20723 | -8.9 |
| 462 | CMNPD28505 | -8.9 |
| 463 | CMNPD30210 | -8.9 |
| 464 | CMNPD24356 | -8.9 |
| 465 | CMNPD10963 | -8.9 |
| 466 | CMNPD27280 | -8.9 |
| 467 | CMNPD18662 | -8.9 |
| 468 | CMNPD24329 | -8.8 |
| 469 | CMNPD28408 | -8.8 |
| 470 | CMNPD24436 | -8.8 |
| 471 | CMNPD21892 | -8.8 |
| 472 | CMNPD23162 | -8.8 |
| 473 | CMNPD30078 | -8.8 |
| 474 | CMNPD20757 | -8.8 |
| 475 | CMNPD1748 | -8.8 |
| 476 | CMNPD18904 | -8.8 |
| 477 | CMNPD27156 | -8.8 |
| 478 | CMNPD17583 | -8.8 |
| 479 | CMNPD17551 | -8.8 |
| 480 | CMNPD17546 | -8.8 |
| 481 | CMNPD27175 | -8.8 |
| 482 | CMNPD15696 | -8.8 |
| 483 | CMNPD14688 | -8.8 |
| 484 | CMNPD28534 | -8.8 |
| 485 | CMNPD25787 | -8.8 |
| 486 | CMNPD26406 | -8.8 |
| 487 | CMNPD17578 | -8.8 |
| 488 | CMNPD27139 | -8.8 |
| 489 | CMNPD18608 | -8.8 |
| 490 | CMNPD30045 | -8.8 |
| 491 | CMNPD20755 | -8.8 |
| 492 | CMNPD19716 | -8.8 |
| 493 | CMNPD8019 | -8.8 |
| 494 | CMNPD30124 | -8.8 |
| 495 | CMNPD21898 | -8.8 |
| 496 | CMNPD28481 | -8.8 |
| 497 | CMNPD4130 | -8.8 |
| 498 | CMNPD25804 | -8.8 |
| 499 | CMNPD28590 | -8.8 |
| 500 | CMNPD20778 | -8.8 |
| 501 | CMNPD13191 | -8.8 |
| 502 | CMNPD13185 | -8.8 |
| 503 | CMNPD24461 | -8.8 |
| 504 | CMNPD30001 | -8.8 |
| 505 | CMNPD3798 | -8.8 |
| 506 | CMNPD30050 | -8.8 |
| 507 | CMNPD24377 | -8.7 |
| 508 | CMNPD28509 | -8.7 |
| 509 | CMNPD27221 | -8.7 |
| 510 | CMNPD28512 | -8.7 |
| 511 | CMNPD19723 | -8.7 |
| 512 | CMNPD25784 | -8.7 |
| 513 | CMNPD19737 | -8.7 |
| 514 | CMNPD19956 | -8.7 |
| 515 | CMNPD24349 | -8.7 |
| 516 | CMNPD20867 | -8.7 |
| 517 | CMNPD25829 | -8.7 |
| 518 | CMNPD9460 | -8.7 |
| 519 | CMNPD3603 | -8.7 |
| 520 | CMNPD25780 | -8.7 |
| 521 | CMNPD24368 | -8.7 |
| 522 | CMNPD17553 | -8.7 |
| 523 | CMNPD15697 | -8.7 |
| 524 | CMNPD23266 | -8.7 |
| 525 | CMNPD30066 | -8.7 |
| 526 | CMNPD27125 | -8.7 |
| 527 | CMNPD7992 | -8.7 |
| 528 | CMNPD16554 | -8.7 |
| 529 | CMNPD28605 | -8.7 |
| 530 | CMNPD27231 | -8.7 |
| 531 | CMNPD19898 | -8.7 |
| 532 | CMNPD17574 | -8.7 |
| 533 | CMNPD23293 | -8.7 |
| 534 | CMNPD24351 | -8.7 |
| 535 | CMNPD23206 | -8.7 |
| 536 | CMNPD20692 | -8.7 |
| 537 | CMNPD25731 | -8.7 |
| 538 | CMNPD28431 | -8.7 |
| 539 | CMNPD16547 | -8.7 |
| 540 | CMNPD27106 | -8.7 |
| 541 | CMNPD20699 | -8.7 |
| 542 | CMNPD28482 | -8.7 |
| 543 | CMNPD20716 | -8.7 |
| 544 | CMNPD30104 | -8.7 |
| 545 | CMNPD19693 | -8.7 |
| 546 | CMNPD23285 | -8.7 |
| 547 | CMNPD20762 | -8.7 |
| 548 | CMNPD15699 | -8.7 |
| 549 | CMNPD17547 | -8.7 |
| 550 | CMNPD30090 | -8.7 |
| 551 | CMNPD30147 | -8.7 |
| 552 | CMNPD27182 | -8.7 |
| 553 | CMNPD27121 | -8.7 |
| 554 | CMNPD28591 | -8.7 |
| 555 | CMNPD19715 | -8.7 |
| 556 | CMNPD28483 | -8.7 |
| 557 | CMNPD21945 | -8.7 |
| 558 | CMNPD19738 | -8.7 |
| 559 | CMNPD16551 | -8.7 |
| 560 | CMNPD10966 | -8.7 |
| 561 | CMNPD27186 | -8.7 |
| 562 | CMNPD19687 | -8.6 |
| 563 | CMNPD21064 | -8.6 |
| 564 | CMNPD27193 | -8.6 |
| 565 | CMNPD30034 | -8.6 |
| 566 | CMNPD27778 | -8.6 |
| 567 | CMNPD10904 | -8.6 |
| 568 | CMNPD19701 | -8.6 |
| 569 | CMNPD21886 | -8.6 |
| 570 | CMNPD28486 | -8.6 |
| 571 | CMNPD27109 | -8.6 |
| 572 | CMNPD27777 | -8.6 |
| 573 | CMNPD24365 | -8.6 |
| 574 | CMNPD29998 | -8.6 |
| 575 | CMNPD325 | -8.6 |
| 576 | CMNPD21885 | -8.6 |
| 577 | CMNPD26404 | -8.6 |
| 578 | CMNPD20718 | -8.6 |
| 579 | CMNPD4648 | -8.6 |
| 580 | CMNPD14695 | -8.6 |
| 581 | CMNPD30055 | -8.6 |
| 582 | CMNPD30061 | -8.6 |
| 583 | CMNPD19721 | -8.6 |
| 584 | CMNPD19733 | -8.6 |
| 585 | CMNPD19714 | -8.6 |
| 586 | CMNPD24429 | -8.6 |
| 587 | CMNPD20767 | -8.6 |
| 588 | CMNPD24428 | -8.6 |
| 589 | CMNPD27278 | -8.6 |
| 590 | CMNPD24405 | -8.6 |
| 591 | CMNPD22292 | -8.6 |
| 592 | CMNPD2188 | -8.6 |
| 593 | CMNPD3602 | -8.6 |
| 594 | CMNPD23303 | -8.6 |
| 595 | CMNPD27168 | -8.6 |
| 596 | CMNPD29954 | -8.6 |
| 597 | CMNPD18663 | -8.6 |
| 598 | CMNPD23159 | -8.6 |
| 599 | CMNPD13190 | -8.6 |
| 600 | CMNPD25742 | -8.6 |
| 601 | CMNPD24456 | -8.6 |
| 602 | CMNPD26412 | -8.6 |
| 603 | CMNPD24501 | -8.6 |
| 604 | CMNPD24462 | -8.6 |
| 605 | CMNPD27791 | -8.6 |
| 606 | CMNPD28643 | -8.6 |
| 607 | CMNPD25794 | -8.6 |
| 608 | CMNPD7988 | -8.6 |
| 609 | CMNPD18623 | -8.6 |
| 610 | CMNPD23222 | -8.6 |
| 611 | CMNPD24426 | -8.6 |
| 612 | CMNPD13996 | -8.6 |
| 613 | CMNPD28476 | -8.6 |
| 614 | CMNPD30054 | -8.6 |
| 615 | CMNPD19727 | -8.6 |
| 616 | CMNPD28628 | -8.6 |
| 617 | CMNPD23236 | -8.6 |
| 618 | CMNPD28633 | -8.6 |
| 619 | CMNPD25747 | -8.6 |
| 620 | CMNPD30105 | -8.6 |
| 621 | CMNPD20725 | -8.6 |
| 622 | CMNPD28453 | -8.6 |
| 623 | CMNPD28446 | -8.6 |
| 624 | CMNPD24434 | -8.6 |
| 625 | CMNPD19917 | -8.6 |
| 626 | CMNPD25879 | -8.6 |
| 627 | CMNPD30106 | -8.6 |
| 628 | CMNPD30025 | -8.6 |
| 629 | CMNPD24392 | -8.6 |
| 630 | CMNPD25777 | -8.6 |
| 631 | CMNPD28553 | -8.6 |
| 632 | CMNPD19728 | -8.6 |
| 633 | CMNPD16564 | -8.6 |
| 634 | CMNPD24380 | -8.5 |
| 635 | CMNPD30060 | -8.5 |
| 636 | CMNPD24494 | -8.5 |
| 637 | CMNPD27281 | -8.5 |
| 638 | CMNPD15800 | -8.5 |
| 639 | CMNPD28572 | -8.5 |
| 640 | CMNPD4125 | -8.5 |
| 641 | CMNPD23163 | -8.5 |
| 642 | CMNPD27127 | -8.5 |
| 643 | CMNPD24433 | -8.5 |
| 644 | CMNPD21887 | -8.5 |
| 645 | CMNPD17550 | -8.5 |
| 646 | CMNPD3147 | -8.5 |
| 647 | CMNPD27107 | -8.5 |
| 648 | CMNPD21890 | -8.5 |
| 649 | CMNPD19722 | -8.5 |
| 650 | CMNPD17580 | -8.5 |
| 651 | CMNPD20680 | -8.5 |
| 652 | CMNPD23244 | -8.5 |
| 653 | CMNPD9401 | -8.5 |
| 654 | CMNPD24370 | -8.5 |
| 655 | CMNPD24473 | -8.5 |
| 656 | CMNPD30159 | -8.5 |
| 657 | CMNPD23230 | -8.5 |
| 658 | CMNPD30059 | -8.5 |
| 659 | CMNPD24379 | -8.5 |
| 660 | CMNPD4127 | -8.5 |
| 661 | CMNPD29999 | -8.5 |
| 662 | CMNPD25858 | -8.5 |
| 663 | CMNPD14777 | -8.5 |
| 664 | CMNPD17552 | -8.5 |
| 665 | CMNPD25881 | -8.5 |
| 666 | CMNPD28407 | -8.5 |
| 667 | CMNPD24431 | -8.5 |
| 668 | CMNPD30137 | -8.5 |
| 669 | CMNPD9462 | -8.5 |
| 670 | CMNPD15705 | -8.5 |
| 671 | CMNPD17548 | -8.5 |
| 672 | CMNPD21965 | -8.5 |
| 673 | CMNPD24487 | -8.5 |
| 674 | CMNPD27174 | -8.5 |
| 675 | CMNPD19724 | -8.5 |
| 676 | CMNPD17560 | -8.5 |
| 677 | CMNPD27237 | -8.5 |
| 678 | CMNPD30190 | -8.5 |
| 679 | CMNPD30214 | -8.5 |
| 680 | CMNPD28552 | -8.5 |
| 681 | CMNPD28551 | -8.5 |
| 682 | CMNPD25732 | -8.5 |
| 683 | CMNPD18688 | -8.5 |
| 684 | CMNPD30086 | -8.5 |
| 685 | CMNPD28479 | -8.5 |
| 686 | CMNPD13189 | -8.5 |
| 687 | CMNPD19713 | -8.5 |
| 688 | CMNPD24432 | -8.5 |
| 689 | CMNPD15694 | -8.5 |
| 690 | CMNPD27183 | -8.5 |
| 691 | CMNPD20710 | -8.5 |
| 692 | CMNPD28465 | -8.5 |
| 693 | CMNPD30063 | -8.5 |
| 694 | CMNPD5857 | -8.4 |
| 695 | CMNPD28635 | -8.4 |
| 696 | CMNPD27177 | -8.4 |
| 697 | CMNPD30101 | -8.4 |
| 698 | CMNPD25730 | -8.4 |
| 699 | CMNPD19709 | -8.4 |
| 700 | CMNPD21977 | -8.4 |
| 701 | CMNPD24373 | -8.4 |
| 702 | CMNPD28477 | -8.4 |
| 703 | CMNPD20724 | -8.4 |
| 704 | CMNPD19720 | -8.4 |
| 705 | CMNPD27130 | -8.4 |
| 706 | CMNPD11660 | -8.4 |
| 707 | CMNPD28634 | -8.4 |
| 708 | CMNPD30221 | -8.4 |
| 709 | CMNPD21888 | -8.4 |
| 710 | CMNPD23233 | -8.4 |
| 711 | CMNPD24495 | -8.4 |
| 712 | CMNPD10984 | -8.4 |
| 713 | CMNPD25882 | -8.4 |
| 714 | CMNPD30165 | -8.4 |
| 715 | CMNPD18634 | -8.4 |
| 716 | CMNPD25748 | -8.4 |
| 717 | CMNPD17604 | -8.4 |
| 718 | CMNPD30219 | -8.4 |
| 719 | CMNPD26411 | -8.4 |
| 720 | CMNPD27131 | -8.4 |
| 721 | CMNPD23268 | -8.4 |
| 722 | CMNPD30065 | -8.4 |
| 723 | CMNPD30064 | -8.4 |
| 724 | CMNPD27171 | -8.4 |
| 725 | CMNPD23264 | -8.4 |
| 726 | CMNPD27191 | -8.4 |
| 727 | CMNPD23251 | -8.4 |
| 728 | CMNPD28535 | -8.4 |
| 729 | CMNPD20742 | -8.4 |
| 730 | CMNPD20682 | -8.4 |
| 731 | CMNPD22320 | -8.4 |
| 732 | CMNPD27150 | -8.4 |
| 733 | CMNPD21913 | -8.4 |
| 734 | CMNPD28671 | -8.4 |
| 735 | CMNPD28472 | -8.4 |
| 736 | CMNPD12380 | -8.4 |
| 737 | CMNPD24344 | -8.4 |
| 738 | CMNPD30084 | -8.4 |
| 739 | CMNPD30071 | -8.4 |
| 740 | CMNPD27217 | -8.4 |
| 741 | CMNPD27132 | -8.4 |
| 742 | CMNPD24347 | -8.4 |
| 743 | CMNPD24977 | -8.4 |
| 744 | CMNPD24353 | -8.4 |
| 745 | CMNPD29955 | -8.4 |
| 746 | CMNPD25750 | -8.4 |
| 747 | CMNPD18668 | -8.4 |
| 748 | CMNPD16735 | -8.4 |
| 749 | CMNPD13968 | -8.4 |
| 750 | CMNPD8750 | -8.4 |
| 751 | CMNPD25857 | -8.4 |
| 752 | CMNPD22308 | -8.4 |
| 753 | CMNPD24425 | -8.4 |
| 754 | CMNPD28459 | -8.4 |
| 755 | CMNPD15678 | -8.4 |
| 756 | CMNPD27232 | -8.3 |
| 757 | CMNPD20726 | -8.3 |
| 758 | CMNPD19920 | -8.3 |
| 759 | CMNPD15682 | -8.3 |
| 760 | CMNPD17543 | -8.3 |
| 761 | CMNPD20779 | -8.3 |
| 762 | CMNPD24496 | -8.3 |
| 763 | CMNPD24476 | -8.3 |
| 764 | CMNPD4126 | -8.3 |
| 765 | CMNPD326 | -8.3 |
| 766 | CMNPD8748 | -8.3 |
| 767 | CMNPD17528 | -8.3 |
| 768 | CMNPD4647 | -8.3 |
| 769 | CMNPD11664 | -8.3 |
| 770 | CMNPD16569 | -8.3 |
| 771 | CMNPD19654 | -8.3 |
| 772 | CMNPD30166 | -8.3 |
| 773 | CMNPD21912 | -8.3 |
| 774 | CMNPD23267 | -8.3 |
| 775 | CMNPD24399 | -8.3 |
| 776 | CMNPD15680 | -8.3 |
| 777 | CMNPD19688 | -8.3 |
| 778 | CMNPD18622 | -8.3 |
| 779 | CMNPD24430 | -8.3 |
| 780 | CMNPD22319 | -8.3 |
| 781 | CMNPD15683 | -8.3 |
| 782 | CMNPD24330 | -8.3 |
| 783 | CMNPD28614 | -8.3 |
| 784 | CMNPD7230 | -8.3 |
| 785 | CMNPD5858 | -8.3 |
| 786 | CMNPD21928 | -8.3 |
| 787 | CMNPD27773 | -8.3 |
| 788 | CMNPD25751 | -8.3 |
| 789 | CMNPD21978 | -8.3 |
| 790 | CMNPD24455 | -8.3 |
| 791 | CMNPD23235 | -8.3 |
| 792 | CMNPD30085 | -8.3 |
| 793 | CMNPD30206 | -8.3 |
| 794 | CMNPD23231 | -8.3 |
| 795 | CMNPD17575 | -8.3 |
| 796 | CMNPD24468 | -8.3 |
| 797 | CMNPD15677 | -8.3 |
| 798 | CMNPD20743 | -8.3 |
| 799 | CMNPD28441 | -8.3 |
| 800 | CMNPD27187 | -8.3 |
| 801 | CMNPD25856 | -8.3 |
| 802 | CMNPD30062 | -8.3 |
| 803 | CMNPD23160 | -8.3 |
| 804 | CMNPD20745 | -8.3 |
| 805 | CMNPD23296 | -8.3 |
| 806 | CMNPD17572 | -8.3 |
| 807 | CMNPD24407 | -8.3 |
| 808 | CMNPD27230 | -8.3 |
| 809 | CMNPD21891 | -8.3 |
| 810 | CMNPD30047 | -8.3 |
| 811 | CMNPD30189 | -8.3 |
| 812 | CMNPD23279 | -8.3 |
| 813 | CMNPD17584 | -8.3 |
| 814 | CMNPD28629 | -8.3 |
| 815 | CMNPD15671 | -8.3 |
| 816 | CMNPD27797 | -8.3 |
| 817 | CMNPD7987 | -8.3 |
| 818 | CMNPD18625 | -8.3 |
| 819 | CMNPD27108 | -8.3 |
| 820 | CMNPD28471 | -8.3 |
| 821 | CMNPD30171 | -8.2 |
| 822 | CMNPD24371 | -8.2 |
| 823 | CMNPD17544 | -8.2 |
| 824 | CMNPD19683 | -8.2 |
| 825 | CMNPD27184 | -8.2 |
| 826 | CMNPD19719 | -8.2 |
| 827 | CMNPD30056 | -8.2 |
| 828 | CMNPD29956 | -8.2 |
| 829 | CMNPD17577 | -8.2 |
| 830 | CMNPD30051 | -8.2 |
| 831 | CMNPD8737 | -8.2 |
| 832 | CMNPD20737 | -8.2 |
| 833 | CMNPD21893 | -8.2 |
| 834 | CMNPD25883 | -8.2 |
| 835 | CMNPD24507 | -8.2 |
| 836 | CMNPD30053 | -8.2 |
| 837 | CMNPD3606 | -8.2 |
| 838 | CMNPD13186 | -8.2 |
| 839 | CMNPD19694 | -8.2 |
| 840 | CMNPD25885 | -8.2 |
| 841 | CMNPD28655 | -8.2 |
| 842 | CMNPD23223 | -8.2 |
| 843 | CMNPD28606 | -8.2 |
| 844 | CMNPD20677 | -8.2 |
| 845 | CMNPD10889 | -8.2 |
| 846 | CMNPD15672 | -8.2 |
| 847 | CMNPD21026 | -8.2 |
| 848 | CMNPD27234 | -8.2 |
| 849 | CMNPD20765 | -8.2 |
| 850 | CMNPD25855 | -8.2 |
| 851 | CMNPD21067 | -8.2 |
| 852 | CMNPD24393 | -8.2 |
| 853 | CMNPD7991 | -8.2 |
| 854 | CMNPD24502 | -8.2 |
| 855 | CMNPD20727 | -8.2 |
| 856 | CMNPD24463 | -8.2 |
| 857 | CMNPD25791 | -8.2 |
| 858 | CMNPD332 | -8.2 |
| 859 | CMNPD27105 | -8.2 |
| 860 | CMNPD28537 | -8.2 |
| 861 | CMNPD24976 | -8.2 |
| 862 | CMNPD30057 | -8.2 |
| 863 | CMNPD15674 | -8.2 |
| 864 | CMNPD28442 | -8.2 |
| 865 | CMNPD19735 | -8.2 |
| 866 | CMNPD25818 | -8.2 |
| 867 | CMNPD28630 | -8.2 |
| 868 | CMNPD28514 | -8.2 |
| 869 | CMNPD19686 | -8.2 |
| 870 | CMNPD25819 | -8.2 |
| 871 | CMNPD22293 | -8.2 |
| 872 | CMNPD24404 | -8.2 |
| 873 | CMNPD23240 | -8.2 |
| 874 | CMNPD5860 | -8.2 |
| 875 | CMNPD23227 | -8.2 |
| 876 | CMNPD17576 | -8.2 |
| 877 | CMNPD24956 | -8.2 |
| 878 | CMNPD28632 | -8.2 |
| 879 | CMNPD24418 | -8.2 |
| 880 | CMNPD19924 | -8.2 |
| 881 | CMNPD24361 | -8.2 |
| 882 | CMNPD29997 | -8.2 |
| 883 | CMNPD24427 | -8.2 |
| 884 | CMNPD28532 | -8.2 |
| 885 | CMNPD30091 | -8.1 |
| 886 | CMNPD28669 | -8.1 |
| 887 | CMNPD10188 | -8.1 |
| 888 | CMNPD14681 | -8.1 |
| 889 | CMNPD24523 | -8.1 |
| 890 | CMNPD27236 | -8.1 |
| 891 | CMNPD23281 | -8.1 |
| 892 | CMNPD5304 | -8.1 |
| 893 | CMNPD25735 | -8.1 |
| 894 | CMNPD19927 | -8.1 |
| 895 | CMNPD25790 | -8.1 |
| 896 | CMNPD27162 | -8.1 |
| 897 | CMNPD15684 | -8.1 |
| 898 | CMNPD27157 | -8.1 |
| 899 | CMNPD23288 | -8.1 |
| 900 | CMNPD27122 | -8.1 |
| 901 | CMNPD24477 | -8.1 |
| 902 | CMNPD12382 | -8.1 |
| 903 | CMNPD3611 | -8.1 |
| 904 | CMNPD28652 | -8.1 |
| 905 | CMNPD13893 | -8.1 |
| 906 | CMNPD2196 | -8.1 |
| 907 | CMNPD25853 | -8.1 |
| 908 | CMNPD30158 | -8.1 |
| 909 | CMNPD28558 | -8.1 |
| 910 | CMNPD28595 | -8.1 |
| 911 | CMNPD15673 | -8.1 |
| 912 | CMNPD24475 | -8.1 |
| 913 | CMNPD28644 | -8.1 |
| 914 | CMNPD30186 | -8.1 |
| 915 | CMNPD19906 | -8.1 |
| 916 | CMNPD21923 | -8.1 |
| 917 | CMNPD19650 | -8.1 |
| 918 | CMNPD24423 | -8.1 |
| 919 | CMNPD17529 | -8.1 |
| 920 | CMNPD28627 | -8.1 |
| 921 | CMNPD24528 | -8.1 |
| 922 | CMNPD27115 | -8.1 |
| 923 | CMNPD27246 | -8.1 |
| 924 | CMNPD19717 | -8.1 |
| 925 | CMNPD28541 | -8.1 |
| 926 | CMNPD25867 | -8.1 |
| 927 | CMNPD28649 | -8.1 |
| 928 | CMNPD28410 | -8.1 |
| 929 | CMNPD30185 | -8.1 |
| 930 | CMNPD10116 | -8.1 |
| 931 | CMNPD19669 | -8.1 |
| 932 | CMNPD24435 | -8.1 |
| 933 | CMNPD23149 | -8.1 |
| 934 | CMNPD10190 | -8.1 |
| 935 | CMNPD27123 | -8.1 |
| 936 | CMNPD5856 | -8.1 |
| 937 | CMNPD10907 | -8.1 |
| 938 | CMNPD27188 | -8.1 |
| 939 | CMNPD28515 | -8.1 |
| 940 | CMNPD20784 | -8.1 |
| 941 | CMNPD20700 | -8.1 |
| 942 | CMNPD23205 | -8.1 |
| 943 | CMNPD29948 | -8.1 |
| 944 | CMNPD24372 | -8.1 |
| 945 | CMNPD344 | -8.1 |
| 946 | CMNPD16575 | -8.1 |
| 947 | CMNPD24362 | -8.1 |
| 948 | CMNPD1024 | -8.1 |
| 949 | CMNPD15690 | -8.1 |
| 950 | CMNPD25843 | -8.1 |
| 951 | CMNPD27133 | -8.1 |
| 952 | CMNPD20756 | -8.1 |
| 953 | CMNPD27185 | -8.1 |
| 954 | CMNPD28478 | -8.1 |
| 955 | CMNPD15670 | -8.1 |
| 956 | CMNPD11663 | -8.1 |
| 957 | CMNPD12468 | -8.1 |
| 958 | CMNPD25820 | -8 |
| 959 | CMNPD25792 | -8 |
| 960 | CMNPD13899 | -8 |
| 961 | CMNPD1747 | -8 |
| 962 | CMNPD17527 | -8 |
| 963 | CMNPD18658 | -8 |
| 964 | CMNPD10191 | -8 |
| 965 | CMNPD28598 | -8 |
| 966 | CMNPD2195 | -8 |
| 967 | CMNPD24388 | -8 |
| 968 | CMNPD18687 | -8 |
| 969 | CMNPD27233 | -8 |
| 970 | CMNPD27120 | -8 |
| 971 | CMNPD17587 | -8 |
| 972 | CMNPD13901 | -8 |
| 973 | CMNPD30218 | -8 |
| 974 | CMNPD29950 | -8 |
| 975 | CMNPD28673 | -8 |
| 976 | CMNPD25826 | -8 |
| 977 | CMNPD24472 | -8 |
| 978 | CMNPD17737 | -8 |
| 979 | CMNPD13900 | -8 |
| 980 | CMNPD28458 | -8 |
| 981 | CMNPD27116 | -8 |
| 982 | CMNPD16703 | -8 |
| 983 | CMNPD25889 | -8 |
| 984 | CMNPD10179 | -8 |
| 985 | CMNPD19926 | -8 |
| 986 | CMNPD20673 | -8 |
| 987 | CMNPD24486 | -8 |
| 988 | CMNPD12383 | -8 |
| 989 | CMNPD28518 | -8 |
| 990 | CMNPD24419 | -8 |
| 991 | CMNPD18659 | -8 |
| 992 | CMNPD24421 | -8 |
| 993 | CMNPD25840 | -8 |
| 994 | CMNPD21946 | -8 |
| 995 | CMNPD28440 | -8 |
| 996 | CMNPD10107 | -8 |
| 997 | CMNPD13178 | -8 |
| 998 | CMNPD239 | -8 |
| 999 | CMNPD20694 | -8 |
| 1000 | CMNPD11711 | -8 |
| 1001 | CMNPD28580 | -8 |
| 1002 | CMNPD24422 | -8 |
| 1003 | CMNPD26403 | -8 |
| 1004 | CMNPD30123 | -8 |
| 1005 | CMNPD21039 | -8 |
| 1006 | CMNPD24474 | -8 |
| 1007 | CMNPD23203 | -8 |
| 1008 | CMNPD18902 | -8 |
| 1009 | CMNPD14696 | -8 |
| 1010 | CMNPD28526 | -8 |
| 1011 | CMNPD28682 | -8 |
| 1012 | CMNPD23254 | -8 |
| 1013 | CMNPD16545 | -8 |
| 1014 | CMNPD27235 | -8 |
| 1015 | CMNPD14699 | -8 |
| 1016 | CMNPD30211 | -8 |
| 1017 | CMNPD27262 | -8 |
| 1018 | CMNPD16538 | -8 |
| 1019 | CMNPD18657 | -8 |
| 1020 | CMNPD13301 | -8 |
| 1021 | CMNPD28485 | -8 |
| 1022 | CMNPD18635 | -8 |
| 1023 | CMNPD12467 | -8 |
| 1024 | CMNPD330 | -8 |
| 1025 | CMNPD3607 | -7.9 |
| 1026 | CMNPD26407 | -7.9 |
| 1027 | CMNPD13979 | -7.9 |
| 1028 | CMNPD17526 | -7.9 |
| 1029 | CMNPD13967 | -7.9 |
| 1030 | CMNPD323 | -7.9 |
| 1031 | CMNPD9390 | -7.9 |
| 1032 | CMNPD16539 | -7.9 |
| 1033 | CMNPD23200 | -7.9 |
| 1034 | CMNPD13202 | -7.9 |
| 1035 | CMNPD21982 | -7.9 |
| 1036 | CMNPD6535 | -7.9 |
| 1037 | CMNPD13969 | -7.9 |
| 1038 | CMNPD333 | -7.9 |
| 1039 | CMNPD12469 | -7.9 |
| 1040 | CMNPD28653 | -7.9 |
| 1041 | CMNPD2190 | -7.9 |
| 1042 | CMNPD30170 | -7.9 |
| 1043 | CMNPD20766 | -7.9 |
| 1044 | CMNPD22303 | -7.9 |
| 1045 | CMNPD6539 | -7.9 |
| 1046 | CMNPD13973 | -7.9 |
| 1047 | CMNPD24957 | -7.9 |
| 1048 | CMNPD25810 | -7.9 |
| 1049 | CMNPD30000 | -7.9 |
| 1050 | CMNPD30093 | -7.9 |
| 1051 | CMNPD30174 | -7.9 |
| 1052 | CMNPD16544 | -7.9 |
| 1053 | CMNPD13270 | -7.9 |
| 1054 | CMNPD8752 | -7.9 |
| 1055 | CMNPD19922 | -7.9 |
| 1056 | CMNPD17741 | -7.9 |
| 1057 | CMNPD10903 | -7.9 |
| 1058 | CMNPD14697 | -7.9 |
| 1059 | CMNPD8745 | -7.9 |
| 1060 | CMNPD1745 | -7.9 |
| 1061 | CMNPD14689 | -7.9 |
| 1062 | CMNPD10967 | -7.9 |
| 1063 | CMNPD28474 | -7.9 |
| 1064 | CMNPD20754 | -7.9 |
| 1065 | CMNPD13188 | -7.9 |
| 1066 | CMNPD20760 | -7.9 |
| 1067 | CMNPD24500 | -7.9 |
| 1068 | CMNPD8751 | -7.9 |
| 1069 | CMNPD20761 | -7.9 |
| 1070 | CMNPD14690 | -7.9 |
| 1071 | CMNPD27282 | -7.9 |
| 1072 | CMNPD30070 | -7.9 |
| 1073 | CMNPD24498 | -7.9 |
| 1074 | CMNPD331 | -7.9 |
| 1075 | CMNPD25811 | -7.9 |
| 1076 | CMNPD24512 | -7.9 |
| 1077 | CMNPD30204 | -7.9 |
| 1078 | CMNPD18661 | -7.9 |
| 1079 | CMNPD16563 | -7.9 |
| 1080 | CMNPD24378 | -7.9 |
| 1081 | CMNPD21915 | -7.9 |
| 1082 | CMNPD27240 | -7.9 |
| 1083 | CMNPD27211 | -7.9 |
| 1084 | CMNPD29929 | -7.9 |
| 1085 | CMNPD27784 | -7.9 |
| 1086 | CMNPD7231 | -7.9 |
| 1087 | CMNPD18879 | -7.9 |
| 1088 | CMNPD8754 | -7.9 |
| 1089 | CMNPD29935 | -7.9 |
| 1090 | CMNPD25851 | -7.9 |
| 1091 | CMNPD4129 | -7.9 |
| 1092 | CMNPD28631 | -7.9 |
| 1093 | CMNPD19736 | -7.9 |
| 1094 | CMNPD17753 | -7.9 |
| 1095 | CMNPD10890 | -7.9 |
| 1096 | CMNPD4645 | -7.9 |
| 1097 | CMNPD24391 | -7.9 |
| 1098 | CMNPD10110 | -7.9 |
| 1099 | CMNPD10108 | -7.9 |
| 1100 | CMNPD311 | -7.8 |
| 1101 | CMNPD23204 | -7.8 |
| 1102 | CMNPD25850 | -7.8 |
| 1103 | CMNPD16570 | -7.8 |
| 1104 | CMNPD24968 | -7.8 |
| 1105 | CMNPD24973 | -7.8 |
| 1106 | CMNPD23144 | -7.8 |
| 1107 | CMNPD21069 | -7.8 |
| 1108 | CMNPD8788 | -7.8 |
| 1109 | CMNPD13997 | -7.8 |
| 1110 | CMNPD10178 | -7.8 |
| 1111 | CMNPD27158 | -7.8 |
| 1112 | CMNPD21975 | -7.8 |
| 1113 | CMNPD22306 | -7.8 |
| 1114 | CMNPD27113 | -7.8 |
| 1115 | CMNPD25842 | -7.8 |
| 1116 | CMNPD19668 | -7.8 |
| 1117 | CMNPD12389 | -7.8 |
| 1118 | CMNPD14712 | -7.8 |
| 1119 | CMNPD12488 | -7.8 |
| 1120 | CMNPD23178 | -7.8 |
| 1121 | CMNPD28473 | -7.8 |
| 1122 | CMNPD28461 | -7.8 |
| 1123 | CMNPD13266 | -7.8 |
| 1124 | CMNPD28610 | -7.8 |
| 1125 | CMNPD29976 | -7.8 |
| 1126 | CMNPD24396 | -7.8 |
| 1127 | CMNPD17530 | -7.8 |
| 1128 | CMNPD9397 | -7.8 |
| 1129 | CMNPD19899 | -7.8 |
| 1130 | CMNPD315 | -7.8 |
| 1131 | CMNPD30234 | -7.8 |
| 1132 | CMNPD24411 | -7.8 |
| 1133 | CMNPD27253 | -7.8 |
| 1134 | CMNPD29949 | -7.8 |
| 1135 | CMNPD238 | -7.8 |
| 1136 | CMNPD28665 | -7.8 |
| 1137 | CMNPD19711 | -7.8 |
| 1138 | CMNPD7239 | -7.8 |
| 1139 | CMNPD27117 | -7.8 |
| 1140 | CMNPD23180 | -7.8 |
| 1141 | CMNPD10886 | -7.8 |
| 1142 | CMNPD6560 | -7.8 |
| 1143 | CMNPD24412 | -7.8 |
| 1144 | CMNPD24346 | -7.8 |
| 1145 | CMNPD11657 | -7.8 |
| 1146 | CMNPD14675 | -7.8 |
| 1147 | CMNPD23229 | -7.8 |
| 1148 | CMNPD15693 | -7.8 |
| 1149 | CMNPD28409 | -7.8 |
| 1150 | CMNPD27785 | -7.8 |
| 1151 | CMNPD28447 | -7.8 |
| 1152 | CMNPD7270 | -7.8 |
| 1153 | CMNPD20736 | -7.8 |
| 1154 | CMNPD9459 | -7.8 |
| 1155 | CMNPD8786 | -7.8 |
| 1156 | CMNPD13254 | -7.8 |
| 1157 | CMNPD28530 | -7.8 |
| 1158 | CMNPD9388 | -7.8 |
| 1159 | CMNPD4128 | -7.8 |
| 1160 | CMNPD10887 | -7.8 |
| 1161 | CMNPD10115 | -7.8 |
| 1162 | CMNPD28462 | -7.8 |
| 1163 | CMNPD2193 | -7.8 |
| 1164 | CMNPD28579 | -7.8 |
| 1165 | CMNPD28674 | -7.8 |
| 1166 | CMNPD24516 | -7.8 |
| 1167 | CMNPD22304 | -7.8 |
| 1168 | CMNPD5303 | -7.8 |
| 1169 | CMNPD23255 | -7.8 |
| 1170 | CMNPD21028 | -7.7 |
| 1171 | CMNPD23292 | -7.7 |
| 1172 | CMNPD28432 | -7.7 |
| 1173 | CMNPD17562 | -7.7 |
| 1174 | CMNPD18677 | -7.7 |
| 1175 | CMNPD20775 | -7.7 |
| 1176 | CMNPD11658 | -7.7 |
| 1177 | CMNPD28529 | -7.7 |
| 1178 | CMNPD23609 | -7.7 |
| 1179 | CMNPD28484 | -7.7 |
| 1180 | CMNPD23146 | -7.7 |
| 1181 | CMNPD19674 | -7.7 |
| 1182 | CMNPD12391 | -7.7 |
| 1183 | CMNPD16559 | -7.7 |
| 1184 | CMNPD10109 | -7.7 |
| 1185 | CMNPD23145 | -7.7 |
| 1186 | CMNPD27242 | -7.7 |
| 1187 | CMNPD10970 | -7.7 |
| 1188 | CMNPD23241 | -7.7 |
| 1189 | CMNPD24493 | -7.7 |
| 1190 | CMNPD20763 | -7.7 |
| 1191 | CMNPD22307 | -7.7 |
| 1192 | CMNPD16556 | -7.7 |
| 1193 | CMNPD29957 | -7.7 |
| 1194 | CMNPD10114 | -7.7 |
| 1195 | CMNPD1761 | -7.7 |
| 1196 | CMNPD23174 | -7.7 |
| 1197 | CMNPD30220 | -7.7 |
| 1198 | CMNPD22294 | -7.7 |
| 1199 | CMNPD28608 | -7.7 |
| 1200 | CMNPD25727 | -7.7 |
| 1201 | CMNPD23143 | -7.7 |
| 1202 | CMNPD30058 | -7.7 |
| 1203 | CMNPD23175 | -7.7 |
| 1204 | CMNPD28460 | -7.7 |
| 1205 | CMNPD2194 | -7.7 |
| 1206 | CMNPD24342 | -7.7 |
| 1207 | CMNPD10964 | -7.7 |
| 1208 | CMNPD25737 | -7.7 |
| 1209 | CMNPD14676 | -7.7 |
| 1210 | CMNPD25841 | -7.7 |
| 1211 | CMNPD27788 | -7.7 |
| 1212 | CMNPD27787 | -7.7 |
| 1213 | CMNPD29982 | -7.7 |
| 1214 | CMNPD25838 | -7.7 |
| 1215 | CMNPD30203 | -7.7 |
| 1216 | CMNPD25837 | -7.7 |
| 1217 | CMNPD24352 | -7.7 |
| 1218 | CMNPD28437 | -7.7 |
| 1219 | CMNPD26421 | -7.7 |
| 1220 | CMNPD13199 | -7.7 |
| 1221 | CMNPD25822 | -7.7 |
| 1222 | CMNPD29961 | -7.7 |
| 1223 | CMNPD28593 | -7.7 |
| 1224 | CMNPD24340 | -7.7 |
| 1225 | CMNPD1746 | -7.7 |
| 1226 | CMNPD20701 | -7.7 |
| 1227 | CMNPD20674 | -7.7 |
| 1228 | CMNPD28444 | -7.7 |
| 1229 | CMNPD21922 | -7.7 |
| 1230 | CMNPD5333 | -7.7 |
| 1231 | CMNPD18682 | -7.7 |
| 1232 | CMNPD23617 | -7.7 |
| 1233 | CMNPD17740 | -7.7 |
| 1234 | CMNPD7240 | -7.7 |
| 1235 | CMNPD25813 | -7.7 |
| 1236 | CMNPD13976 | -7.7 |
| 1237 | CMNPD13194 | -7.7 |
| 1238 | CMNPD21947 | -7.7 |
| 1239 | CMNPD24359 | -7.7 |
| 1240 | CMNPD28445 | -7.7 |
| 1241 | CMNPD22295 | -7.7 |
| 1242 | CMNPD25726 | -7.7 |
| 1243 | CMNPD16555 | -7.7 |
| 1244 | CMNPD23610 | -7.7 |
| 1245 | CMNPD23185 | -7.7 |
| 1246 | CMNPD18886 | -7.7 |
| 1247 | CMNPD28687 | -7.7 |
| 1248 | CMNPD16558 | -7.7 |
| 1249 | CMNPD10961 | -7.6 |
| 1250 | CMNPD16537 | -7.6 |
| 1251 | CMNPD30164 | -7.6 |
| 1252 | CMNPD10968 | -7.6 |
| 1253 | CMNPD5852 | -7.6 |
| 1254 | CMNPD28508 | -7.6 |
| 1255 | CMNPD28438 | -7.6 |
| 1256 | CMNPD28611 | -7.6 |
| 1257 | CMNPD29960 | -7.6 |
| 1258 | CMNPD23302 | -7.6 |
| 1259 | CMNPD30069 | -7.6 |
| 1260 | CMNPD16553 | -7.6 |
| 1261 | CMNPD24453 | -7.6 |
| 1262 | CMNPD25833 | -7.6 |
| 1263 | CMNPD2569 | -7.6 |
| 1264 | CMNPD19667 | -7.6 |
| 1265 | CMNPD27124 | -7.6 |
| 1266 | CMNPD15691 | -7.6 |
| 1267 | CMNPD25888 | -7.6 |
| 1268 | CMNPD20866 | -7.6 |
| 1269 | CMNPD28636 | -7.6 |
| 1270 | CMNPD29981 | -7.6 |
| 1271 | CMNPD24506 | -7.6 |
| 1272 | CMNPD22291 | -7.6 |
| 1273 | CMNPD24452 | -7.6 |
| 1274 | CMNPD16591 | -7.6 |
| 1275 | CMNPD23243 | -7.6 |
| 1276 | CMNPD27249 | -7.6 |
| 1277 | CMNPD30023 | -7.6 |
| 1278 | CMNPD17566 | -7.6 |
| 1279 | CMNPD5853 | -7.6 |
| 1280 | CMNPD23182 | -7.6 |
| 1281 | CMNPD24357 | -7.6 |
| 1282 | CMNPD30099 | -7.6 |
| 1283 | CMNPD26399 | -7.6 |
| 1284 | CMNPD18684 | -7.6 |
| 1285 | CMNPD27223 | -7.6 |
| 1286 | CMNPD23238 | -7.6 |
| 1287 | CMNPD16568 | -7.6 |
| 1288 | CMNPD12392 | -7.6 |
| 1289 | CMNPD16536 | -7.6 |
| 1290 | CMNPD25887 | -7.6 |
| 1291 | CMNPD24503 | -7.6 |
| 1292 | CMNPD28672 | -7.6 |
| 1293 | CMNPD24360 | -7.6 |
| 1294 | CMNPD23215 | -7.6 |
| 1295 | CMNPD17559 | -7.6 |
| 1296 | CMNPD25847 | -7.6 |
| 1297 | CMNPD19670 | -7.6 |
| 1298 | CMNPD28436 | -7.6 |
| 1299 | CMNPD13193 | -7.6 |
| 1300 | CMNPD24478 | -7.6 |
| 1301 | CMNPD24962 | -7.6 |
| 1302 | CMNPD28435 | -7.6 |
| 1303 | CMNPD30208 | -7.6 |
| 1304 | CMNPD30087 | -7.6 |
| 1305 | CMNPD28681 | -7.6 |
| 1306 | CMNPD29959 | -7.6 |
| 1307 | CMNPD27251 | -7.6 |
| 1308 | CMNPD15686 | -7.6 |
| 1309 | CMNPD25832 | -7.6 |
| 1310 | CMNPD28507 | -7.6 |
| 1311 | CMNPD24864 | -7.6 |
| 1312 | CMNPD18636 | -7.6 |
| 1313 | CMNPD21972 | -7.6 |
| 1314 | CMNPD25808 | -7.6 |
| 1315 | CMNPD30187 | -7.6 |
| 1316 | CMNPD13255 | -7.6 |
| 1317 | CMNPD28457 | -7.6 |
| 1318 | CMNPD30207 | -7.6 |
| 1319 | CMNPD27104 | -7.6 |
| 1320 | CMNPD13196 | -7.6 |
| 1321 | CMNPD24343 | -7.6 |
| 1322 | CMNPD30162 | -7.6 |
| 1323 | CMNPD20702 | -7.6 |
| 1324 | CMNPD7271 | -7.5 |
| 1325 | CMNPD28467 | -7.5 |
| 1326 | CMNPD30156 | -7.5 |
| 1327 | CMNPD25752 | -7.5 |
| 1328 | CMNPD19666 | -7.5 |
| 1329 | CMNPD9399 | -7.5 |
| 1330 | CMNPD25824 | -7.5 |
| 1331 | CMNPD30233 | -7.5 |
| 1332 | CMNPD28599 | -7.5 |
| 1333 | CMNPD24444 | -7.5 |
| 1334 | CMNPD25758 | -7.5 |
| 1335 | CMNPD25728 | -7.5 |
| 1336 | CMNPD22263 | -7.5 |
| 1337 | CMNPD17731 | -7.5 |
| 1338 | CMNPD21882 | -7.5 |
| 1339 | CMNPD9402 | -7.5 |
| 1340 | CMNPD19710 | -7.5 |
| 1341 | CMNPD30157 | -7.5 |
| 1342 | CMNPD25764 | -7.5 |
| 1343 | CMNPD340 | -7.5 |
| 1344 | CMNPD25823 | -7.5 |
| 1345 | CMNPD23177 | -7.5 |
| 1346 | CMNPD8787 | -7.5 |
| 1347 | CMNPD19672 | -7.5 |
| 1348 | CMNPD30155 | -7.5 |
| 1349 | CMNPD27789 | -7.5 |
| 1350 | CMNPD28565 | -7.5 |
| 1351 | CMNPD18672 | -7.5 |
| 1352 | CMNPD10182 | -7.5 |
| 1353 | CMNPD30119 | -7.5 |
| 1354 | CMNPD23192 | -7.5 |
| 1355 | CMNPD30092 | -7.5 |
| 1356 | CMNPD15692 | -7.5 |
| 1357 | CMNPD27148 | -7.5 |
| 1358 | CMNPD23214 | -7.5 |
| 1359 | CMNPD16542 | -7.5 |
| 1360 | CMNPD8739 | -7.5 |
| 1361 | CMNPD11715 | -7.5 |
| 1362 | CMNPD24505 | -7.5 |
| 1363 | CMNPD9466 | -7.5 |
| 1364 | CMNPD20685 | -7.5 |
| 1365 | CMNPD14691 | -7.5 |
| 1366 | CMNPD24485 | -7.5 |
| 1367 | CMNPD30161 | -7.5 |
| 1368 | CMNPD28625 | -7.5 |
| 1369 | CMNPD20689 | -7.5 |
| 1370 | CMNPD19901 | -7.5 |
| 1371 | CMNPD30205 | -7.5 |
| 1372 | CMNPD13980 | -7.5 |
| 1373 | CMNPD7274 | -7.5 |
| 1374 | CMNPD25839 | -7.5 |
| 1375 | CMNPD16541 | -7.5 |
| 1376 | CMNPD19698 | -7.5 |
| 1377 | CMNPD20672 | -7.5 |
| 1378 | CMNPD28626 | -7.5 |
| 1379 | CMNPD10180 | -7.5 |
| 1380 | CMNPD24504 | -7.5 |
| 1381 | CMNPD20705 | -7.5 |
| 1382 | CMNPD19651 | -7.5 |
| 1383 | CMNPD17571 | -7.5 |
| 1384 | CMNPD25724 | -7.5 |
| 1385 | CMNPD20749 | -7.5 |
| 1386 | CMNPD14692 | -7.5 |
| 1387 | CMNPD18683 | -7.5 |
| 1388 | CMNPD8744 | -7.5 |
| 1389 | CMNPD12462 | -7.5 |
| 1390 | CMNPD13972 | -7.5 |
| 1391 | CMNPD24383 | -7.5 |
| 1392 | CMNPD25845 | -7.5 |
| 1393 | CMNPD24488 | -7.5 |
| 1394 | CMNPD23171 | -7.5 |
| 1395 | CMNPD8022 | -7.5 |
| 1396 | CMNPD30128 | -7.5 |
| 1397 | CMNPD26415 | -7.5 |
| 1398 | CMNPD27250 | -7.5 |
| 1399 | CMNPD18644 | -7.5 |
| 1400 | CMNPD30163 | -7.5 |
| 1401 | CMNPD20675 | -7.5 |
| 1402 | CMNPD24420 | -7.5 |
| 1403 | CMNPD30107 | -7.5 |
| 1404 | CMNPD30215 | -7.5 |
| 1405 | CMNPD18611 | -7.5 |
| 1406 | CMNPD27790 | -7.5 |
| 1407 | CMNPD27207 | -7.5 |
| 1408 | CMNPD22289 | -7.4 |
| 1409 | CMNPD13902 | -7.4 |
| 1410 | CMNPD10102 | -7.4 |
| 1411 | CMNPD20734 | -7.4 |
| 1412 | CMNPD17581 | -7.4 |
| 1413 | CMNPD25761 | -7.4 |
| 1414 | CMNPD3608 | -7.4 |
| 1415 | CMNPD8738 | -7.4 |
| 1416 | CMNPD13198 | -7.4 |
| 1417 | CMNPD21934 | -7.4 |
| 1418 | CMNPD15698 | -7.4 |
| 1419 | CMNPD27238 | -7.4 |
| 1420 | CMNPD23202 | -7.4 |
| 1421 | CMNPD22302 | -7.4 |
| 1422 | CMNPD13203 | -7.4 |
| 1423 | CMNPD11722 | -7.4 |
| 1424 | CMNPD30232 | -7.4 |
| 1425 | CMNPD30118 | -7.4 |
| 1426 | CMNPD23189 | -7.4 |
| 1427 | CMNPD23186 | -7.4 |
| 1428 | CMNPD23179 | -7.4 |
| 1429 | CMNPD24445 | -7.4 |
| 1430 | CMNPD20706 | -7.4 |
| 1431 | CMNPD20774 | -7.4 |
| 1432 | CMNPD30102 | -7.4 |
| 1433 | CMNPD18871 | -7.4 |
| 1434 | CMNPD19918 | -7.4 |
| 1435 | CMNPD5301 | -7.4 |
| 1436 | CMNPD28601 | -7.4 |
| 1437 | CMNPD3609 | -7.4 |
| 1438 | CMNPD14787 | -7.4 |
| 1439 | CMNPD7993 | -7.4 |
| 1440 | CMNPD16540 | -7.4 |
| 1441 | CMNPD13903 | -7.4 |
| 1442 | CMNPD28654 | -7.4 |
| 1443 | CMNPD16543 | -7.4 |
| 1444 | CMNPD319 | -7.4 |
| 1445 | CMNPD26405 | -7.4 |
| 1446 | CMNPD13207 | -7.4 |
| 1447 | CMNPD23154 | -7.4 |
| 1448 | CMNPD27215 | -7.4 |
| 1449 | CMNPD322 | -7.4 |
| 1450 | CMNPD18681 | -7.4 |
| 1451 | CMNPD13282 | -7.4 |
| 1452 | CMNPD24358 | -7.4 |
| 1453 | CMNPD11654 | -7.4 |
| 1454 | CMNPD28439 | -7.4 |
| 1455 | CMNPD20721 | -7.4 |
| 1456 | CMNPD19673 | -7.4 |
| 1457 | CMNPD24961 | -7.4 |
| 1458 | CMNPD28600 | -7.4 |
| 1459 | CMNPD26409 | -7.4 |
| 1460 | CMNPD24339 | -7.4 |
| 1461 | CMNPD23299 | -7.4 |
| 1462 | CMNPD24333 | -7.4 |
| 1463 | CMNPD17726 | -7.4 |
| 1464 | CMNPD21974 | -7.4 |
| 1465 | CMNPD30098 | -7.4 |
| 1466 | CMNPD21966 | -7.4 |
| 1467 | CMNPD21070 | -7.4 |
| 1468 | CMNPD17565 | -7.4 |
| 1469 | CMNPD27248 | -7.4 |
| 1470 | CMNPD9398 | -7.4 |
| 1471 | CMNPD25836 | -7.4 |
| 1472 | CMNPD24865 | -7.4 |
| 1473 | CMNPD28680 | -7.4 |
| 1474 | CMNPD29947 | -7.4 |
| 1475 | CMNPD7272 | -7.4 |
| 1476 | CMNPD16732 | -7.4 |
| 1477 | CMNPD28594 | -7.4 |
| 1478 | CMNPD13195 | -7.4 |
| 1479 | CMNPD10181 | -7.4 |
| 1480 | CMNPD24513 | -7.4 |
| 1481 | CMNPD8790 | -7.4 |
| 1482 | CMNPD27206 | -7.4 |
| 1483 | CMNPD30038 | -7.4 |
| 1484 | CMNPD9456 | -7.4 |
| 1485 | CMNPD17579 | -7.4 |
| 1486 | CMNPD28546 | -7.3 |
| 1487 | CMNPD18563 | -7.3 |
| 1488 | CMNPD24457 | -7.3 |
| 1489 | CMNPD26408 | -7.3 |
| 1490 | CMNPD13971 | -7.3 |
| 1491 | CMNPD25844 | -7.3 |
| 1492 | CMNPD29983 | -7.3 |
| 1493 | CMNPD17738 | -7.3 |
| 1494 | CMNPD23275 | -7.3 |
| 1495 | CMNPD10962 | -7.3 |
| 1496 | CMNPD3610 | -7.3 |
| 1497 | CMNPD18630 | -7.3 |
| 1498 | CMNPD18646 | -7.3 |
| 1499 | CMNPD24410 | -7.3 |
| 1500 | CMNPD10965 | -7.3 |


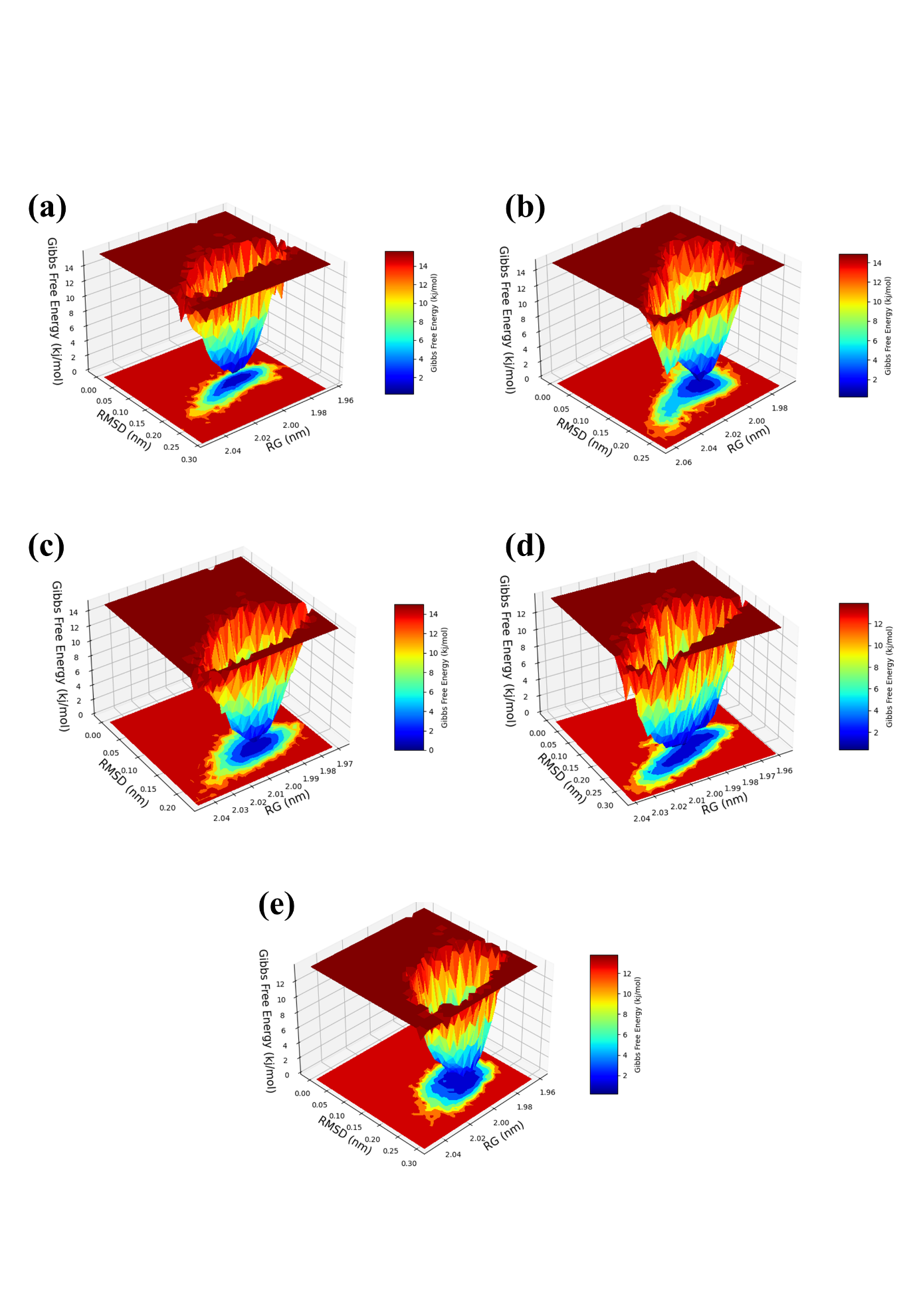


**Figure S1 –** 3D structure analysis of four selected compounds in complex with the protein (a) CMNPD27283, (b) CMNPD19660, (c) CMNPD27166, and (d) CMNPD24402 and (e) control (Q0J)

**
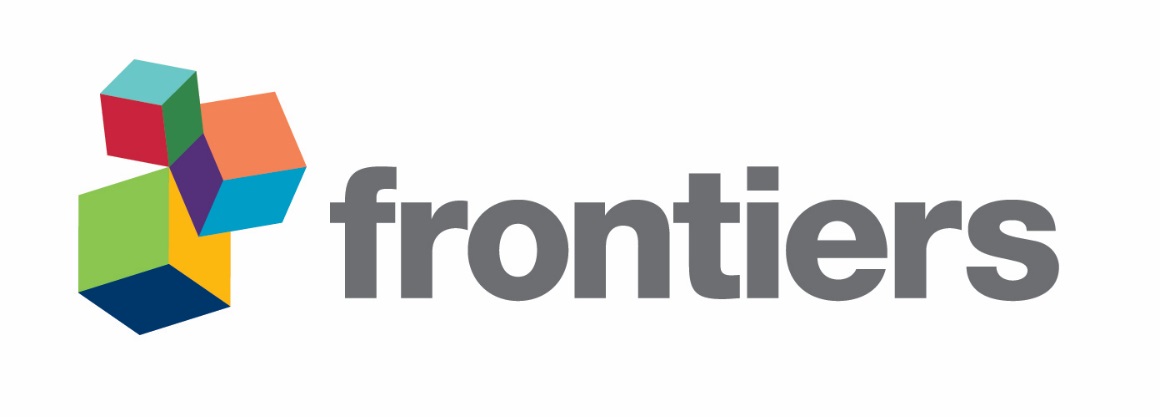
**
